# Unveiling Scaling Laws of Parameter Identifiability and Uncertainty Quantification in Data-Driven Biological Modeling

**Published:** 2026-02-24

**Authors:** Shun Wang, Wenrui Hao

**Affiliations:** 1 Department of Mathematics, Penn State University, University Park, Pennsylvania, USA.

**Keywords:** Parameter Identifiability, Uncertainty Quantification, Data-driven Modeling, Asymptotic Analysis

## Abstract

Integrating high-dimensional biological data into data-driven mechanistic modeling requires rigorous practical identifiability to ensure interpretability and generalizability. However, coordinate identifiability analysis often suffers from numerical instabilities near singular local minimizers. We present a computational framework that uncovers fundamental scaling laws governing practical identifiability through asymptotic analysis. By synthesizing Fisher information with perturbed Hessian matrices, we establish a hierarchical approach to quantify coordinate identifiability and inform uncertainty quantification within non-identifiable subspaces across different orders. Supported by rigorous mathematical analysis and validated on synthetic and real-world data, our framework was applied to HIV-host dynamics and spatiotemporal amyloid-β propagation. These applications demonstrate the framework’s efficiency in elucidating critical mechanisms underlying HIV diagnostics and Alzheimer’s disease progression. In the era of large-scale mechanistic digital twins, our framework provides the scaling laws for data-driven modeling in terms of both parameter identifiability and uncertainty, ensuring that data-driven inferences are grounded in verifiable biological reality.

## Teaser

Leveraging Parameter Identifiability Scaling Laws to Build Robust Data-Driven Biological Models.

## Introduction

Incorporating high-dimensional biological data into data-driven mechanistic modeling has become a robust framework for elucidating complex biological processes and forecasting disease trajectories across multiple scales. At the subcellular and cellular levels, integrating single-cell transcriptomics with kinetic rate equations—particularly through the lens of RNA velocity (1)—enables reconstruction of latent developmental trajectories by modeling transitions from unspliced to spliced mRNA (2, 3, 4, 5, 6). At the mesoscopic tissue scale, spatial proteomics and fluorescent labeling of the tumor microenvironment (7, 8, 9, 7) support the development of spatiotemporal interaction models that capture the biophysical constraints governing immune cell infiltration and tumor-immune dynamics (10,11). At the macroscopic clinical level, longitudinal biomarker datasets such as those from the Alzheimer’s Disease Neuroimaging Initiative (ADNI) facilitate cascade modeling (12, 13), mapping the stereo-typical deposition patterns of Amyloid-β and Tau proteins across the temporal progression of neurodegeneration (14, 15).

Despite these advancements, translating phenomenological observations into predictive clinical modeling remains hindered by challenges in parameter identifiability (16) and model uncertainty (17). A fundamental prerequisite for the reliability of data-driven frameworks is determining which model parameters can be uniquely inferred from the limited, noisy, and sparse biological data (18). Moreover, rigorously quantifying uncertainty distributions is essential for assessing the confidence and reliability of model predictions (19). Addressing these modeling bottlenecks is therefore critical for the robust translation of data-driven models into actionable precision medicine tools and clinical decision-support systems (20).

First, parameter (practical) identifiability determines whether model parameters can be uniquely resolved from specific experimental observations. The profile likelihood method has long served as the gold standard for this assessment (16, 21, 22, 23, 24, 25, 26). Nevertheless, applying traditional approaches—particularly the profile likelihood method—to high-dimensional biological systems is often infeasible due to prohibitive computational costs. Recently, a systematic computational framework has been proposed that formalizes practical identifiability on a rigorous mathematical foundation, introduces a high-efficiency metric to alleviate computational burdens, and bridges the gap between the theoretical definition of identifiability and its practical assessment (27). However, the current aproaches face significant challenges when resolving parameters characterized by relatively flat likelihood profiles. While the conventional profile likelihood approach can still resolve such parameters using a calibrated statistical threshold, developing quantitative indices that rigorously characterize these low-curvature cases remains an open challenge. This “flatness” is not merely a numerical artifact but a recurring feature across multiple biological scales—including morphogen gradients (26, 28), virus-host dynamics (29, 30), and taxol-treated cancer models (31, 32)—where the relative insensitivity of the objective function to parameter perturbations necessitates a more nuanced approach to threshold selection to ensure predictive reliability (33). These observations strongly suggest the existence of underlying scaling laws of parameter identifiability that can systematically characterize such behaviors.

Second, model uncertainty in complex biological systems is fundamentally linked to practical non-identifiability, where insufficient information to constrain parameter values propagates as ambiguity in model outputs (34, 35). While existing uncertainty quantification (UQ) methods assess how non-identifiable parameters influence predictions (27), they currently lack the capability to quantify how uncertainty and confidence intervals scale with the intrinsic identifiability of each parameter. Establishing a rigorous connection between practical identifiability and uncertainty propagation is therefore essential for reliable predictive modeling.

In this paper, building upon the recent developed parameter identifiablity framework (27), we present a rigorous computational framework that reveals the scaling law of practical identifiability. This approach significantly enhances the detection of higher-order identifiability, effectively characterizing parameter identifiability where traditional profile likelihood methods typically fail due to numerical insensitivity on ‘flat’ likelihood manifolds. Furthermore, we establish a robust UQ protocol that explicitly accounts for higher-order practically non-identifiable parameters. Supported by formal mathematical proofs and validated against the simulation data, our framework was applied to HIV host-virus dynamics and the spatiotemporal distribution of amyloid-β(Aβ) in the brain. These applications demonstrate the framework’s capacity to elucidate critical biological mechanisms underlying HIV therapeutic responses and the progression of Alzheimer’s disease.

## Results

### Overview of the Computational Approach to Parameter Identifiability Scaling Laws

We consider a general model φ(t,θ), where t denotes the independent input variable (e.g., time) and $\theta \in {\mathbb{R}}^{k}$ is the parameter vector. The model architecture is versatile, encompassing explicit functional forms (e.g., neural networks) or solutions to complex differential equations, with observable variables h(φ(t,θ)) mapped to experimental measurements ${\left\{t_{i},{\hat{h}}_{i}\right\}}_{i=1}^{N}$ collected at discrete time points.

Using the least-squares objective $l(h(t,\theta ),\hat{h})=\sum_{i=1}^{N}{\left\|h\left(t_{i},\theta \right)-{\hat{h}}_{i}\right\|}_{2}^{2}$, we obtain the optimal parameter set $\theta^{*}$ and compute the Fisher Information Matrix (FIM, F) and the perturbed Hessian $\left(H\right)$ (27). Our framework integrates eigenvalue decomposition (EVD) with the Schur complement (SC) to classify parameter identifiability across hierarchical scales. The algorithm is illustrated in Algorithm 1, and a detailed mathematical formulation is provided in Materials and Methods.

We introduce a novel metric, $K_{i}$, to quantify the i-th order of parameter identifiability. The zero-order component, $K_{0}$, recovers traditional parameter identifiability, $K_{0}=\|{\left(I-\left.AA^{†}\right)s\right.}_{i}{\|}_{2}^{2}$, (27), while the first-order metric, $K_{1}$, captures the emergence of flat likelihood profiles when $K_{0}=0$. A detailed analytical formulation of $K_{i}$ is provided in Algorithm 1.

**Figure 1:**
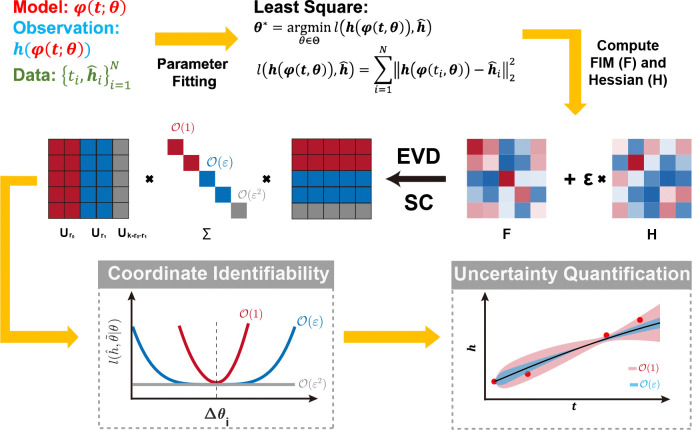
Illustration of the scaling law of parameter identifiablity. Schematic framework of higher-order parameter identifiability analysis integrates eigenvalue decomposition (EVD) and the Schur complement (SC) to categorize parameter identifiability across hierarchical scales. The eigenvalue matrix Σ is color-coded to denote the order of identifiability: red indicates zero-order identifiability [O(1)], blue represents first-order identifiability [O(ε)], and gray corresponds to second-order identifiability $\left[O(\varepsilon^{2})\right]$. Within the eigenvector matrix U, these spectral regimes define specific parameter combinations: zero-order identifiable coordinates ($U_{r_{0}}^{⊤}\theta$, red), first-order identifiable coordinates ($U_{r_{1}}^{⊤}\theta$, blue), and second-order identifiable coordinates ($U_{k-r_{0}-r_{1}}^{⊤}\theta$, gray). The metric $K_{i}$ is employed to determine the higher-order coordinate practical identifiability. Furthermore, the higher-order uncertainty quantification (UQ) framework evaluates the predictive uncertainty originating from non-identifiable subspaces, specifically isolating contributions from zero-order non-identifiable parameters ($U_{k-r_{0}}^{⊤}\theta$, red region) and first-order non-identifiable parameters ($U_{k-r_{0}-r_{1}}^{⊤}\theta$, blue region).

Furthermore, our higher-order uncertainty quantification (UQ) framework evaluates predictive uncertainty arising from non-identifiable subspaces. Specifically, it isolates contributions from zero-order non-identifiable parameters ($U_{k-r_{0}}^{⊤}\theta$, red region) and first-order non-identifiable parameters ($U_{k-r_{0}-r_{1}}^{⊤}\theta$, blue region) in Fig. 1, thereby also characterizing the uncertainty order of the loss function.

### Polynomial Fitting Benchmark Example

To evaluate the accuracy of our framework, we first apply it to a polynomial model defined as

(1) $$h(t;\theta )=\theta_{1}(|t-2.5|-3)+\theta_{2}[(t-1)(t-2)(t-3)(t-4)+1.5]+\frac{1}{2}\theta_{2}^{2}\frac{0.5(t-2)(t-3)(t-4)}{\left(1-2)(1-3)(1-4\right)}\;\;+\theta_{3}[(t-1)(t-2)(t-3)(t-4)+1.0]+\theta_{4}[(t-1)(t-2)(t-3)(t-4)+0.5].$$

We then compute the parameter identifiability using our framework and compare the results with the profile likelihood method (16,36), which serves as a benchmark in asymptotic analysis of parameter identifiability.

**Figure 2:**
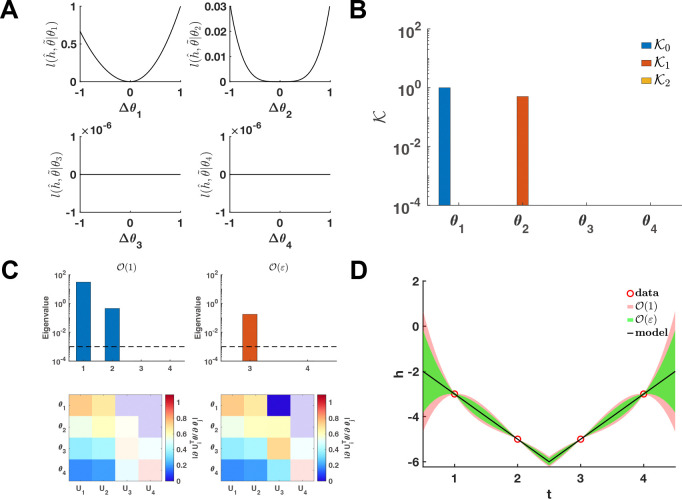
Validation method accuracy in polynomial fitting. **(A)** Coordinate identifiability analysis at $\theta^{*}={\left[2,0,0,0\right]}^{T}$ using the profile likelihood. **(B)** The metrics $K_{i}$ for conducting practical identifiability analysis. **(C)** Eigenvalue of δ-order practical identifiability analysis and heatmap of the eigenvector matrix. The dashed line is the threshold $\epsilon =10^{-3}$. The color bar represents the values of each eigenvector element. The shaded area indicates the eigenvectors corresponding to ε-order non-identifiable parameters. **(D)** UQ from the perturbation to ε-order non-identifiable parameters. Circles represent the synthetic data generated from the polynomial function. The solid line represents the polynomial function with the given parameter values $\theta^{*}$. The red and green shaded regions represent the 95% confidence intervals under zero-order and first-order *ε* perturbations of the non-identifiable parameters, respectively.

Given the parameter set $\theta^{*}$, we employ the profile likelihood method (16, 36) to evaluate the practical identifiability of each parameter in Eq. 1, establishing a benchmark for comparison (Fig. 2a). The conditional loss function $l\left(\hat{h},\tilde{\theta };\theta_{2}\right)$ for parameter $\theta_{2}$ exhibits a degenerate convex profile, characterized by regions of low curvature (flatness), whereas the conditional loss function $l\left(\hat{h},\tilde{\theta };\theta_{1}\right)$ displays sharply peaked convexity (Fig. 2A).

Our proposed method computes the metrics $K_{i}$ to assess the coordinate-wise identifiability of all parameters in Eq. 1. The results show that parameter $\theta_{1}$ possesses zero-order practical identifiability $\left(K_{0}>0\right)$, while parameter $\theta_{2}$ exhibits first-order practical identifiability $\left(K_{0}=0,K_{1}>0\right)$. In contrast, the remaining parameters are non-identifiable $\left(K_{i}\right)$ (Fig. 2B). These classifications are in close agreement with the benchmark results (Fig. 2A).

Notably, if only the zero-order metric $\left(K_{0}\right)$ is considered, parameter $\theta_{2}$ is incorrectly classified as non-identifiable (Fig. 2B); however, the benchmark analysis confirms its practical identifiability (Fig. 2A). This discrepancy highlights the critical necessity of a higher-order practical identifiability framework to resolve parameters that reside within the flat regions of the likelihood surface.

According to the asymptotic analysis of parameter identifiability, the eigenvalues further reveal the practical identifiability of the parameters (Fig. 2C). Specifically, $U_{1}^{⊤}\theta$ and $U_{2}^{⊤}\theta$ exhibit zero-order practical identifiability, whereas $U_{3}^{⊤}\theta$ displays first-order parameter identifiability. In contrast, $U_{4}^{⊤}\theta$ is non-identifiable (Fig. 2C). Using our UQ computational framework, we introduce parameter perturbations and calculate the 95% confidence interval for variations in the dependent variable. As illustrated in Fig. 2D, whether considering the zero-order or first-order non-identifiable subspaces, the resulting confidence intervals vanish at the discrete data points. This convergence indicates that the objective function is locally invariant to perturbations restricted to the non-identifiable parameter manifold, confirming that such variations do not compromise the model’s agreement with empirical observations. Moreover, the uncertainty region derived from the first-order non-identifiable subspace is contained within that generated by the zero-order non-identifiable subspace. This hierarchical nesting of uncertainty bounds provides empirical validation for the theoretical framework established in Theorem 3.

### Higher-Order Parameter Identifiability in the HIV Kinetic Model

**Figure 3:**
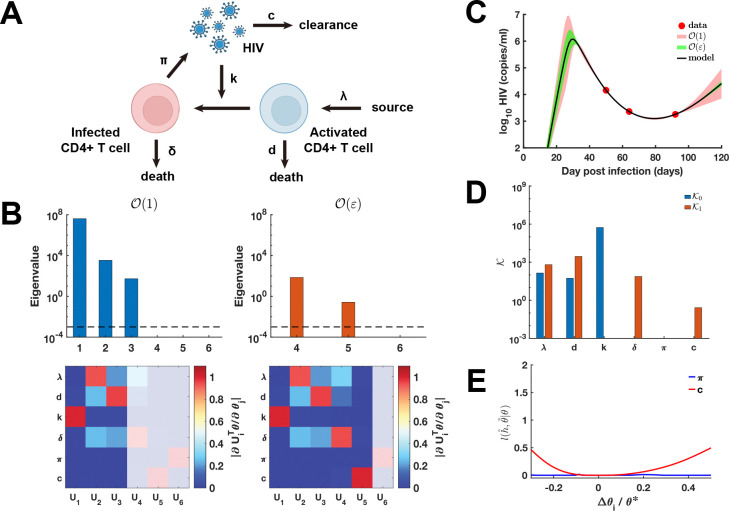
Higher-order parameter identifiability analysis of HIV virus-host dynamics. **(A)** Schematic of the HIV infection model. **(B)** Eigenvalues from the *ε*-order practical identifiability analysis and heatmap of the corresponding eigenvector matrix. The dashed line indicates the threshold $\epsilon =10^{-3}$. The color bar represents the magnitude of each eigenvector element, and the shaded area highlights eigenvectors corresponding to ε-order non-identifiable parameters. **(C)** Uncertainty quantification (UQ) from perturbations of ε-order non-identifiable parameters. Circles represent clinical log-transformed plasma HIV concentrations (38), and the solid line shows reconstructed dynamics using $\theta^{*}$ from Table S1. The red and green shaded regions denote 95% confidence intervals for zero-order and first-order ε perturbations, respectively. **(D)** Metrics $K_{i}$ used for higher-order practical identifiability analysis. **(E)** Coordinate-wise identifiability analysis for parameters π and c using the profile likelihood method.

To investigate the scaling laws of parameter identifiability in data-driven biological modeling, we apply our framework to a canonical HIV infection model (37, 38). This system, governed by a set of ordinary differential equations (ODEs) (see section “HIV host-virus dynamics” in the Supplementary Materials), incorporates six fundamental parameters that characterize key viral kinetic processes (Fig. 3A; see Table S1 for detailed parameter descriptions). The observable variable is defined as the log-transformed plasma HIV concentration,

$$h(t;\theta )=\log_{10}V(t;\theta ),$$

consistent with clinical longitudinal measurements from HIV-infected patients (38).

Following the determination of the optimal parameter set $\theta^{*}$ via the least-squares objective defined in Eqs. 2–3, we conducted an asymptotic analysis of parameter identifiability. Our results reveal a hierarchical structure in the parameter space: the eigen-coordinates $U_{1}^{⊤}\theta$, $U_{2}^{⊤}\theta$, and $U_{3}^{⊤}\theta$ exhibit zero-order practical identifiability, whereas $U_{4}^{⊤}\theta$ and $U_{5}^{⊤}\theta$ display first-order practical identifiability (Fig. 3B). In contrast, $U_{6}^{⊤}\theta$ is non-identifiable. Using the $K_{i}$ metric, we find that parameters λ, d, and k exhibit zero-order practical identifiability, while δ and c show first-order practical identifiability (Fig. 3D). Parameter π is classified as non-identifiable. To validate these findings, we performed a profile likelihood analysis for c and π; the resulting profiles are in close agreement with the $K_{i}$-based classification, confirming the sensitivity of the metric to varying degrees of parameter resolution (Fig. 3E).

The higher-order UQ analysis reveals that parameters exhibiting zero-order non-identifiability exert a broad influence over both the initial HIV outbreak and the subsequent phases of viral relapse. Conversely, parameters characterized by first-order non-identifiability primarily modulate the early stages of infection, with their impact diminishing as the system progresses. These results demonstrate that the hierarchical order of practical identifiability effectively maps the architecture of predictive uncertainty onto the temporal evolution of HIV virus-host dynamics, suggesting that higher-order practical identifiability is essential for delineating which phases of the infection—from initial outbreak to long-term relapse—are most susceptible to parameter-induced ambiguity.

### Hierarchical Parameter Identifiability in the Amyloid-β Aggregation Spatiotemporal Model

**Figure 4:**
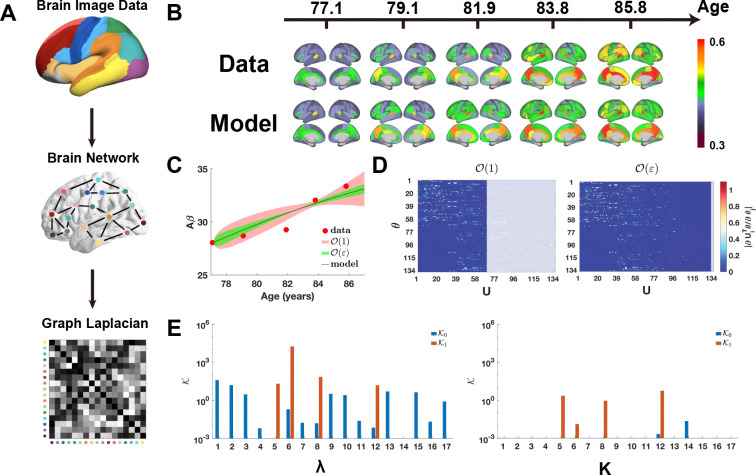
Hierarchical parameter identifiability of Amyloid-β(Aβ) spatiotemporal dynamics. **(A)** Image preprocessing pipeline for constructing the graph Laplacian matrix. **(B)** Reconstruction of Aβ spatiotemporal dynamics using the network-based PDE model. The colorbar represents the standardized uptake value ratios (SUVRs) of Aβ. **(C)** Uncertainty quantification (UQ) from perturbations of ε-order non-identifiable parameters. Circles denote observed Aβ SUVRs, and the solid line shows reconstructed dynamics using $\theta^{*}$ from Tables S2–S8. The red and green shaded regions represent 95% confidence intervals for zero-order and first-order ε perturbations, respectively. **(D)** Heatmap of the eigenvector matrix. The dashed line indicates the threshold $\epsilon =2\times 10^{-3}$. The color bar represents the magnitude of each eigenvector element, and the shaded area highlights eigenvectors corresponding to ε-order non-identifiable parameters. **(E)** Metrics $K_{i}$ used for higher-order practical identifiability analysis of the first 17 brain regions.

We applied our higher-order practical identifiability framework to investigate the spatiotemporal dynamics of Amyloid-β(Aβ). Using neuroimaging data from the ADNI cohort, we partitioned the brain into 68 cortical regions and constructed a graph Laplacian matrix to represent the structural connectome (Fig. 4A; see section “Spatiotemporal dynamics of *Aβ* on the brain” in the Supplementary Materials for details). These connectivity patterns were incorporated into a networked PDE model (Eq. S76). In contrast to the low-dimensional HIV models discussed previously, the parameter dimensionality of this spatiotemporal system exceeds the computational capacity of traditional profile likelihood methods, highlighting the need for a more scalable analytical approach.

Following the determination of the optimal parameter set $\theta^{*}$ via the least-squares objective defined in Eqs. 2–3, the spatiotemporal Aβ dynamics are accurately reconstructed by the networked PDE model (Fig. 4B). Subsequent asymptotic analysis of practical identifiability reveals a complex spectral hierarchy: roughly half of the eigen-coordinates exhibit zero-order identifiability, while three specific coordinates display first-order identifiability (Figs. 4D and S1A). Notably, such high-dimensional resolution is numerically inaccessible via traditional profile likelihood methods, which fail to resolve fine-grained parameter identifiability in large-scale networked systems. Using the $K_{i}$ metric, we find that the regional production rates, λ, exhibit either zero-order or first-order practical identifiability across cortical regions. In contrast, the parameters governing the local Aβ carrying capacity, K, manifest only first-order practical identifiability or remain non-identifiable (Figs. 4E and S1B). The observed monotonic increase in Aβ accumulation over time (Fig. 4B) is fully consistent with our asymptotic analysis of coordinate-wise practical identifiability.

Finally, we performed a higher-order uncertainty quantification (UQ) analysis, which revealed distinct sensitivity patterns across different orders of parameter identifiability. Eigen-coordinates exhibiting zero-order non-identifiability show vanishing uncertainty only at age 84. In contrast, those characterized by first-order non-identifiability display a broader window of zero uncertainty spanning ages 82 to 84 (Fig. 4C). Notably, this interval aligns with the phase of rapid Aβ accumulation: whereas zero-order UQ identifies only the terminal state at age 84, the first-order UQ analysis captures the entire temporal window of the Aβ outbreak.

## Discussions

Parameter identifiability is a cornerstone of data-driven modeling in biological systems, directly determining the reliability, robustness, and predictive power of model-based inferences. As data-driven approaches gain prominence, rigorous assessment of parameter identifiability has become essential. Empirical datasets are often sparse and noisy, obscuring the underlying parameter landscape and leaving models vulnerable to overfitting stochastic fluctuations rather than capturing the true governing dynamics. While this challenge has received substantial attention, existing methods frequently rely on heuristic criteria or numerical approximations (16, 36, 34), which are computationally intensive and prone to local instabilities. Such limitations can yield misleading conclusions about parameter resolvability, undermining confidence in model predictions. Consequently, establishing a rigorous framework for parameter identifiability has shifted from a peripheral concern to a fundamental mathematical prerequisite for translating data-driven insights into actionable biological knowledge.

In this work, we uncover the fundamental scaling laws of parameter identifiability, as formalized in the definition of practical identifiability (27), by introducing the perturbated Hessian matrix $\left(H\right)$. Within this framework, the infinitesimal parameter ε arises from an experimental perturbation δ, providing a rigorous mathematical basis to quantify the sensitivity mapping between the parameter manifold and empirical observations. We formalize higher-order parameter identifiability in Theorem 1 and introduce a hierarchical series of scales, $K_{i}$, which substantially reduce the computational burden associated with traditional profile likelihood methods. Analytical corollaries (Corollaries 2 and 3) reveal that the emergence of flat likelihood profiles is primarily governed by the first-order metric $K_{1}$, enabling prediction of complex likelihood surface topologies directly from $K_{i}$ without exhaustive numerical optimization.

The higher-order UQ analysis projects predictive uncertainty onto non-identifiable subspaces, establishing a hierarchical containment principle in which higher-order uncertainty manifolds are nested within their zero-order counterparts (Theorem 3). Complementing this geometric insight, the derived uncertainty orders for the loss function (Eqs. 41–42) rigorously quantify the sensitivity of the objective landscape to infinitesimal perturbations in empirical data. Using a benchmark system, we validated these theorems and metrics, showing complete agreement between analytical predictions and numerical landscapes generated via the profile likelihood method (Fig. 2). These results confirm that our asymptotic framework accurately captures higher-order practical identifiability while providing a direct, computationally efficient alternative to exhaustive likelihood scanning.

We validated the hierarchical scaling laws of parameter identifiability using two distinct high-dimensional, data-driven paradigms: nonlinear HIV-host viral dynamics and the spatiotemporal propagation of Aβ in the human brain. In the canonical HIV infection model, higher-order parameter identifiability is prominently observed in the virion production rate (π) of infected CD4+ T cells and the viral clearance rate (c) (Fig. 3D). This higher-order resolution is essential for delineating specific pathophysiological phases of HIV infection governed by distinct parameter subsets (Fig. 3C). Within our networked-PDE framework for Aβ dynamics—a hallmark biomarker of Alzheimer’s disease—higher-order parameter identifiability is evident in the carrying capacity parameters $\left(K\right)$ across individual cortical regions (Fig. 4E). Notably, while zero-order UQ captures only the terminal saturation state, higher-order identifiability successfully resolves the transient phase of rapid Aβ accumulation (Fig. 4C), highlighting its capacity to detect critical transition windows in disease progression.

Although this study focuses on the least-squares objective under the assumption of small residuals, within which the FIM and perturbated Hessian accurately approximate the loss Hessian (Eq. 11), the framework extends naturally to more general objective functions, including cross-entropy or scenarios with large residuals. In these cases, parameter identifiability analysis remains mathematically consistent by substituting the FIM with the Hessian of the generalized loss function and reevaluating the perturbation operator $M_{1}[\delta ]$ accordingly.

In summary, we present a novel framework for higher-order parameter identifiability analysis, grounded in the scaling laws that govern parameter resolvability. By systematically integrating fundamental properties of identifiability with hierarchical analytical metrics and higher-order UQ protocols, our approach establishes a rigorous mathematical bridge between high-dimensional biological models and noisy empirical data. Supported by comprehensive theoretical proofs, the framework delineates the hierarchical structure of practical identifiability across multiple orders of magnitude. As biological modeling increasingly shifts toward large-scale mechanistic digital twins, this higher-order framework provides a critical diagnostic tool to ensure that data-driven insights are translated into robust, actionable, and biologically meaningful knowledge.

## Materials and Methods

First, we introduce the Fisher Information Matrix (FIM) and the perturbed Hessian matrix to define parameter identifiability. Next, we develop a series of asymptotic analysis theorems to rigorously quantify higher-order parameter identifiability and establish the corresponding scaling laws. Finally, we present an uncertainty quantification (UQ) approach to capture how uncertainty arising from higher-order practically non-identifiable parameters propagates into model predictions.

### Practical Identifiability Definition

For the time-series data-driven modeling approach, the loss function $l(h(t,\theta ),\hat{h})$ is defined using the least squares method as follows:

(2) $$l(h(t,\theta ),\hat{h})=\sum_{i=1}^{N}{\left\|h\left(t_{i},\theta \right)-{\hat{h}}_{i}\right\|}_{2}^{2},h\left(t_{i},\theta \right)=h\left(\varphi \left(t_{i},\theta \right)\right)$$

where N is the number of experimental data, $\varphi (t,\theta )\in {\mathbb{R}}^{M}$ denotes the system output with parameter $\theta \in {\mathbb{R}}^{K}$ at the time $t={\left[t_{1},t_{2},\ldots ,t_{N}\right]}^{⊤}\left(\varphi (t,\theta )={\left[\varphi_{1}(t,\theta ),\varphi_{2}(t,\theta ),\cdots ,\varphi_{M}(t,\theta )\right]}^{⊤}\right.$, and $\left.\varphi_{i}(t,\theta )={\left[\varphi_{i}\left(t_{1},\theta \right),\varphi_{i}\left(t_{2},\theta \right),\cdots ,\varphi_{i}\left(t_{N},\theta \right)\right]}^{⊤}\right)$. The experimental observation is denoted as ${\left\{\left(t_{i},{\hat{h}}_{i}\right)\right\}}_{i=1}^{N}$, $\left(\hat{h}={\left[{\hat{h}}_{1},{\hat{h}}_{2},\ldots ,{\hat{h}}_{N}\right]}^{⊤}\right)$, and the continuous differentiable function h(⋅) represents measurable quantities $\left(h(\cdot )\in {\mathbb{R}}^{L}\right)$. The parameters of this system $\theta^{*}$ are given as

(3) $$\theta^{*}=\arg\;\underset{\theta \in \text{Θ}}{\min}\;l(h(t,\theta ),\hat{h})$$

where θ is the parameter space. The parameter of this system $\theta_{\delta }$ for the presence of small perturbation (δ) in measurements is obtained as

(4) $$\theta_{\delta }=\arg\;\underset{\theta \in \text{Θ}}{\min}\;l(h(t,\theta ),\hat{h}-\delta )$$

Herein, the loss function $l(h(t,\theta ),\hat{h}-\delta )$ is hypothesized to be continuous with respect to small perturbation (δ). Our previous work (27) proposed the definition of practical identifiability based on the changes in parameters resulting from measurement perturbations (Eqs. 3–4) as follows:

#### Definition 1.

*The parameter* θ *in* Θ *is practically identifiable if* ∀ε>0, ∃C>0 *such that* $\|\delta \|<\varepsilon \Rightarrow \left\|\theta_{\delta }-\theta^{*}\right\|<C\varepsilon$ *where* $\theta^{*}$ *and* $\theta_{\delta }$ *satisfy Eq. 3 and Eq. 4, respectively*.

### FIM and Hessian Matrix

Using the Taylor extension, The loss $l(h(t,\theta ),\hat{h})$ is approximately expressed as a second-order derivation as follows:

(5) $$\begin{array}{l} l\left(h\left(t,\theta_{\delta }\right);\hat{h}-\delta \right)\approx l\left(h\left(t,\theta^{*}\right);\hat{h}-\delta \right)+{\left(\nabla_{\theta }l\left(h\left(t,\theta^{*}\right);\hat{h}-\delta \right)\right)}^{⊤}\text{Δ}\theta \\ \;+\frac{1}{2}\text{Δ}\theta^{⊤}\left(\nabla_{\theta }^{2}l\left(h\left(\theta^{*}\right);\hat{h}-\delta \right)\right)\text{Δ}\theta . \end{array}$$

The second-order derivation term $\nabla_{\theta }^{2}l\left(h\left(\theta^{*}\right);\hat{h}-\delta \right)$ expands to perturbation δ at δ=0 using Taylor expansion as:

(6) $$\begin{array}{l} \nabla_{\theta }^{2}l\left(h\left(t,\theta^{*}\right);\hat{h}-\delta \right)=\sum_{k=0}^{\infty }\frac{{\left(-1\right)}^{k}}{k!}M_{k}\left[\delta^{⊗k}\right]\\ \;M_{k}\left[\delta^{⊗k}\right]=\sum_{i=1}^{N}{\left(\nabla_{\theta }h\left(t_{i},\theta^{*}\right)\right)}^{⊤}\left(\nabla_{\hat{h}}^{k}\nabla_{h}^{2}l\left(h\left(t_{i},\theta^{*}\right);\hat{h}\right)\right)\left[\delta^{⊗k}\right]\nabla_{\theta }h\left(t_{i},\theta^{*}\right)\\ \;+\sum_{i=1}^{N}\sum_{l=1}^{L}\left(\nabla_{\hat{h}}^{k}\frac{\partial l}{\partial h_{l}}\left(h\left(t_{i},\theta^{*}\right);\hat{h}\right)\right)\left[\delta^{⊗k}\right]\cdot \nabla_{\theta }^{2}h_{l}\left(t_{i},\theta^{*}\right);\\ \;\left(\nabla_{\hat{h}}^{k}\nabla_{\hat{h}}^{2}l\right)\left[\delta^{⊗k}\right]={\left[\sum_{j_{1},\ldots ,j_{k}}\frac{\partial^{k+2}l}{\partial h_{a}\partial h_{b}\partial {\hat{h}}_{j_{1}}\cdots \partial {\hat{h}}_{j_{k}}}\cdot \delta_{j_{1}}\cdots \delta_{j_{k}}\right]}_{a,b=1}^{L};\\ \left(\nabla_{\hat{h}}^{k}\frac{\partial l}{\partial h_{i}}(h,\hat{h})\right)\left[\delta^{⊗k}\right]=\sum_{j_{1},\ldots ,j_{k}=1}^{L}\frac{\partial^{k+1}l}{\partial h_{i}\partial {\hat{h}}_{j_{1}}\cdots \partial {\hat{h}}_{j_{k}}}(h;\hat{h})\cdot \delta_{j_{1}}\cdots \delta_{j_{k}}. \end{array}$$

Since l is the least square loss, we just need to compute the $M_{0}$ and $M_{1}[\delta ]$ because of $M_{k}[\delta ]=0(k\geq 2)$. When k=0, $M_{0}$ is the Hessian matrix as follows:

(7) $$M_{0}=\sum_{i=1}^{N}{\left(\nabla_{\theta }h\left(t_{i},\theta^{*}\right)\right)}^{⊤}\left(\nabla_{h}^{2}l\left(h\left(t_{i},\theta^{*}\right);\hat{h}\right)\right)\nabla_{\theta }h\left(t_{i},\theta^{*}\right)+\sum_{i=1}^{N}\sum_{l=1}^{L}\frac{\partial l}{\partial h_{l}}\left(h\left(t_{i},\theta^{*}\right);\hat{h}\right)\cdot \nabla_{\theta }^{2}h_{l}\left(t_{i},\theta^{*}\right).$$

When k=1, $M_{1}[\delta ]$ is obtained as:

(8) $$M_{1}[\delta ]=\sum_{i=1}^{N}{\left(\nabla_{\theta }h\left(t_{i},\theta^{*}\right)\right)}^{⊤}\left(\nabla_{\hat{h}}\nabla_{h}^{2}l\left(h\left(t_{i},\theta^{*}\right);\hat{h}\right)\right)[\delta ]\nabla_{\theta }h\left(t_{i},\theta^{*}\right)\;\;+\sum_{i=1}^{N}\sum_{l=1}^{L}\left(\nabla_{\hat{h}}\frac{\partial l}{\partial h_{l}}\left(h\left(t_{i},\theta^{*}\right);\hat{h}\right)\right)[\delta ]\cdot \nabla_{\theta }^{2}h_{l}\left(t_{i},\theta^{*}\right)$$

Substitute the L-2 loss l into $M_{0}$ and $M_{1}[\delta ]$, we have

(9) $$\begin{array}{l} \;M_{0}=\sum_{i=1}^{N}{\left(\nabla_{\theta }h\left(t_{i},\theta^{*}\right)\right)}^{⊤}\nabla_{\theta }h\left(t_{i},\theta^{*}\right)+\sum_{i=1}^{N}\sum_{l=1}^{L}\left(h_{l}\left(t_{i},\theta^{*}\right)-{\hat{h}}_{l}\left(t_{i}\right)\right)\cdot \nabla_{\theta }^{2}h_{l}\left(t_{i},\theta^{*}\right)\\ M_{1}[\delta ]=\sum_{i=1}^{N}\sum_{l=1}^{L}\left(-\delta_{l}\right)\cdot \nabla_{\theta }^{2}h_{l}\left(t_{i},\theta^{*}\right), \end{array}$$

and the second-order derivation term $\nabla_{\theta }^{2}l\left(h\left(t,\theta^{*}\right);\hat{h}-\delta \right)$ is obtained as:

(10) $$\begin{array}{l} \nabla_{\theta }^{2}l\left(h\left(t,\theta^{*}\right);\hat{h}-\delta \right)=\sum_{i=1}^{N}{\left(\nabla_{\theta }h\left(t_{i},\theta^{*}\right)\right)}^{⊤}\nabla_{\theta }h\left(t_{i},\theta^{*}\right)+\sum_{i=1}^{N}\sum_{l=1}^{L}\left(-\delta_{l}\right)\cdot \nabla_{\theta }^{2}h_{l}\left(t_{i},\theta^{*}\right)\\ \;+\sum_{i=1}^{N}\sum_{l=1}^{L}\left(h_{l}\left(t_{i},\theta^{*}\right)-{\hat{h}}_{l}\left(t_{i}\right)\right)\cdot \nabla_{\theta }^{2}h_{l}\left(t_{i},\theta^{*}\right). \end{array}$$

Denote the FIM of $l(h(\theta );\hat{h})$ as $F=M_{0}$, and the perturbation Hessian matrix $\sum_{i=1}^{N}\sum_{l=1}^{L}\left(-\delta_{l}\right)$. $\nabla_{\theta }^{2}h_{l}\left(t_{i},\theta^{*}\right)$ is approximately expressed as εH where ‖δ‖<ε and $H=\sum_{i=1}^{N}\sum_{l=1}^{L}\nabla_{\theta }^{2}h_{l}\left(t_{i},\theta^{*}\right)$.

### Asymptotic Analysis of Parameter Identifiability

In general, the model is assumed to fit the data with small residual $\left(\left\|h\left(t;\theta^{*}\right)-\hat{h}(t)\right\|\approx 0\right)$ so that the second-order derivation term is simplified as $\nabla_{\theta }^{2}l\left(h\left(t,\theta^{*}\right);\hat{h}-\delta \right)\approx F+\varepsilon H$. Having demonstrating the relationship between FIM and practical identifiability (27), we utilize Schur Complement Theory to provide an asymptotic analysis framework of practical identifiabilty. We introduce the definition of Schur complement matrix (39) as follows:

#### Definition 2.

*Let* $Q\in {\mathbb{R}}^{k\times k}$ *be a symmetric block matrix partitioned as*

$$Q=\left[\begin{array}{cc} A & B\\ B^{⊤} & C \end{array}\right],\;A\in {\mathbb{R}}^{r\times r},\;C\in {\mathbb{R}}^{\left(k-r)\times (k-r\right)}.$$

*If* A *is invertible, the Schur complement of the block* A *of the matrix* Q *is defined by*

$$S_{A}:=C-B^{⊤}A^{-1}B.$$

*If* C *is invertible, then the Schur complement of the block* C *of the matrix* Q *is defined by*

$$S_{C}:=A-BC^{-1}B^{⊤}.$$

Building upon the theorem that the parameter θ in Θ is practically identifiable if and only if the FIM $F\left(\theta^{*}\right)$ is invertible (27) and noting that both the FIM (F) and the perturbation Hessian matrix (H) are positive semi-definite when $\theta^{*}$ satisfies Eq. 3, we extend this framework to a more generalized formulation of ε–order practical identifiability as stated in in Theorem 1. This extension is achieved by integrating Schur Complement Theory with eigenvalue decomposition (EVD) (39) to rigorously quantify practical identifiability.

#### Theorem 1.

*The matrix* F+εH *can be approximately expressed to third-order of* ε *as*

(11) $$F+\varepsilon H\approx \hat{U}\left[\begin{array}{cccc} {\text{Σ}}_{r_{0}}+B_{0} & & & \\ & \varepsilon \left({\text{Σ}}_{r_{1}}+B_{1}\right) & & \\ & & \varepsilon^{2}\left({\text{Σ}}_{r_{2}}+B_{2}\right) & \\ & & & \varepsilon^{3}S_{3} \end{array}\right]{\hat{U}}^{⊤}$$

*The practically identifiable and non-identifiable parameters at multiple* ε-*order are displayed as:*

1. *Zero-order* ε(O(1))*: the non-identifiable parameter is* $U_{k-r_{0}}^{(0)⊤}\theta$ *and the practically identifiable parameter is* $U_{r_{0}}^{(0)⊤}\theta$;
2. *First-order* ε(O(ε))*: the non-identifiable parameter* $U_{k-\sum_{i=0}^{1}r_{i}}^{(1)⊤}U_{k-r_{0}}^{(0)⊤}\theta$, *and the practically identifiable parameter is* $\left[U_{r_{0}}^{(0)⊤}\theta +Z_{1}^{⊤}U_{k-r_{0}}^{(0)⊤}\theta ;U_{r_{1}}^{(1)⊤}U_{k-r_{0}}^{(0)⊤}\theta \right]$
3. *Second-order* $\varepsilon \left(O\left(\varepsilon^{2}\right)\right)$*: the non-identifiable parameter is* $U_{k-\sum_{i=0}^{2}r_{i}}^{(2)⊤}U_{k-\sum_{i=0}^{1}r_{i}}^{(1)⊤}U_{k-r_{0}}^{(0)⊤}\theta$, *and the practically identifiable parameter is*
$$\left[U_{r_{0}}^{(0)⊤}\theta +Z_{1}^{⊤}U_{k-r_{0}}^{(0)⊤}\theta ;U_{r_{1}}^{(1)⊤}U_{k-r_{0}}^{(0)⊤}\theta +Z_{2}^{⊤}U_{k-r_{o}-r_{1}}^{(1)⊤}U_{k-r_{0}}^{(0)⊤}\theta ;U_{r_{2}}^{(2)⊤}U_{k-\sum_{i=0}^{1}r_{i}}^{(1)⊤}U_{k-r_{0}}^{(0)⊤}\theta \right]$$

Details of the proof including the definitions of matrices in Eq. 11 are shown in the “Proof of Theorem 1” section in Supplementary Materials. Meanwhile, we can obtain a corollary on practical identifiability as below:

#### Corollary 1.

*If* H *is a positive definite matrix in the null space of* F, *i.e.*, H∈null(F)∖{0}, *the parameter* θ *in* Θ *is practically identifiable*.

Details of the proof are shown in the “Proof of Corollary 1” section in Supplementary Materials.

### Asymptotic Analysis of Coordinate Practical Identifiability

Coordinate practical identifiability is defined using the Bayesian posterior likelihood (34, 16, 36) as follows:

#### Definition 3.

*The parameter* θ *is coordinate practically identifiable if the profile likelihood* $PL\left(\hat{h}|\theta_{i}\right)=\min_{\theta_{j\neq i}}[l(\hat{h};\theta )]$ *has a locally unique minimum at* $\theta_{i}^{*}$ *for each parameter coordinate* i.

Since both the FIM (F) and the perturbation Hessian matrix (H) are semi-positive definite under an $L_{2}$ loss regime, the fundamental nature of Definition 3 is as follows: the minimization of loss function $L\left(\theta_{i},\tilde{\theta };\varepsilon \right)=\text{Δ}\theta^{⊤}(F+\varepsilon H)\text{Δ}\theta$ strictly exceeds zero-specially, $\min_{\tilde{\theta }}L\left(\theta_{i},\tilde{\theta };\varepsilon \right)>0$ when the parameter θ is rewritten as $\theta =\left[\tilde{\theta };\theta_{i}\right]$. First, we perform the permutation to the matrix F and H for $\theta =\left[\tilde{\theta };\theta_{i}\right]$ as:

(12) $$\hat{F}=P_{i}^{⊤}FP,\;\hat{H}=P_{i}^{⊤}HP.$$

where $P_{i}=\left[e_{1},e_{2},\cdots ,e_{i-1},e_{k},e_{i+1},\cdots ,e_{k-1},e_{i}\right]$ is the elementary matrix and the vector $e_{i}$ is unit vector. The block matrix for $\hat{F}$ and $\hat{H}$ are rewritten as

(13) $$\hat{F}=\left[\begin{array}{ll} {\hat{F}}_{11} & {\hat{F}}_{21}^{⊤}\\ {\hat{F}}_{21} & {\hat{F}}_{22} \end{array}\right],\;\hat{H}=\left[\begin{array}{ll} {\hat{H}}_{11} & {\hat{H}}_{21}^{⊤}\\ {\hat{H}}_{21} & {\hat{H}}_{22} \end{array}\right]$$

where ${\hat{F}}_{11}$, ${\hat{H}}_{11}\in {\mathbb{R}}^{\left(k-1)\times (k-1\right)}$, ${\hat{F}}_{21}$, ${\hat{H}}_{21}\in {\mathbb{R}}^{k-1}$, ${\hat{F}}_{22}$, ${\hat{H}}_{22}\in \mathbb{R}$. Then, the loss $L\left(\theta_{i},\tilde{\theta };\varepsilon \right)$ is rewritten as

(14) $$L\left(\theta_{i},\tilde{\theta };\varepsilon \right)=a(\varepsilon )\theta_{i}^{2}+2\theta_{i}b^{⊤}(\varepsilon )\tilde{\theta }+{\tilde{\theta }}^{⊤}c(\varepsilon )\tilde{\theta }$$

where

(15) $$a(\varepsilon )={\hat{F}}_{22}+\varepsilon {\hat{H}}_{22},\;b(\varepsilon )={\hat{F}}_{21}+\varepsilon {\hat{H}}_{21},\;c(\varepsilon )={\hat{F}}_{11}+\varepsilon {\hat{H}}_{11}.$$

Then, the minimization of $L\left(\theta_{i},\tilde{\theta };\varepsilon \right)$ to $\tilde{\theta }$ satisfies the necessary condition:

(16) $$2b(\varepsilon )\theta_{i}+2c(\varepsilon )\tilde{\theta }=0,\;\tilde{\theta }=-c{\left(\varepsilon \right)}^{†}b(\varepsilon )\theta_{i}$$

We can obtain the loss $L\left(\theta_{i};\tilde{\theta },\varepsilon \right)$ as

(17) $$L\left(\theta_{i};\tilde{\theta },\varepsilon \right)=\left({\hat{F}}_{22}+\varepsilon {\hat{H}}_{22}-\left({\hat{F}}_{21}+\varepsilon {\hat{H}}_{21}\right){\left({\hat{F}}_{11}+\varepsilon {\hat{H}}_{11}\right)}^{†}{\left({\hat{F}}_{21}+\varepsilon {\hat{H}}_{21}\right)}^{⊤}\right)\theta_{i}^{2}$$

Next, we perform the EVD for matrix ${\hat{F}}_{11}$ and rewrite the matrix ${\hat{H}}_{11}$ as:

(18) $${\hat{F}}_{11}=U\left[\begin{array}{ll} \text{Λ} & 0\\ 0 & 0 \end{array}\right]U^{⊤},\;{\hat{H}}_{11}=U\left[\begin{array}{ll} h_{11} & h_{12}\\ h_{12}^{⊤} & h_{22} \end{array}\right]U^{⊤}$$

When ε is small enough, the inverse of matrix $\text{Λ}+\varepsilon h_{11}$ exists. We can find the lower triangular matrix

(19) $$L=\left[\begin{array}{cc} I & 0\\ \varepsilon h_{12}^{⊤}{\left(\text{Λ}+\varepsilon h_{11}\right)}^{-1} & I \end{array}\right]$$

to make the matrix ${\hat{F}}_{11}+\varepsilon {\hat{H}}_{11}$ as follows:

(20) $${\hat{F}}_{11}+\varepsilon {\hat{H}}_{11}=UL\left[\begin{array}{cc} \text{Λ}+\varepsilon h_{11} & 0\\ 0 & S \end{array}\right]L^{⊤}U^{⊤},\;S=\varepsilon h_{22}-\varepsilon^{2}h_{12}^{⊤}{\left({\text{Λ}}_{r}+\varepsilon h_{11}\right)}^{-1}h_{12}$$

So, we can compute the generalized inverse of ${\hat{F}}_{11}+\varepsilon {\hat{H}}_{11}$ as:

(21) $${\left({\hat{F}}_{11}+\varepsilon {\hat{H}}_{11}\right)}^{†}=U{\left(L^{⊤}\right)}^{-1}\left[\begin{array}{cc} {\left(\text{Λ}+\varepsilon h_{11}\right)}^{-1} & 0\\ 0 & S^{†} \end{array}\right]L^{-1}U^{⊤}\;=U\left[\begin{array}{cc} I & -\varepsilon {\left(\text{Λ}+\varepsilon h_{11}\right)}^{-1}h_{12}\\ 0 & I \end{array}\right]\left[\begin{array}{cc} {\left(\text{Λ}+\varepsilon h_{11}\right)}^{-1} & 0\\ 0 & S^{†} \end{array}\right]\left[\begin{array}{cc} I & 0\\ -\varepsilon {\left(\text{Λ}+\varepsilon h_{11}\right)}^{-1}h_{12} & I \end{array}\right]U^{⊤}\;=U\left[\begin{array}{cc} {\left(\text{Λ}+\varepsilon h_{11}\right)}^{-1}+{\left(\text{Λ}+\varepsilon h_{11}\right)}^{-1}\left(\varepsilon h_{12}\right)S^{†}\left(\varepsilon h_{12}^{⊤}\right){\left(\text{Λ}+\varepsilon h_{11}\right)}^{-1} & -{\left(\text{Λ}+\varepsilon h_{11}\right)}^{-1}\left(\varepsilon h_{12}\right)S^{†}\\ -S^{†}\left(\varepsilon h_{12}^{⊤}\right){\left(\text{Λ}+\varepsilon h_{11}\right)}^{-1} & S^{†} \end{array}\right]U^{⊤}$$

When we just consider the aprroximation using the Neumann series as:

(22) $${\left(\text{Λ}+\varepsilon h_{11}\right)}^{-1}\approx {\text{Λ}}^{-1}-\varepsilon {\text{Λ}}^{-1}h_{11}{\text{Λ}}^{-1}+\varepsilon^{2}{\text{Λ}}^{-1}h_{11}{\text{Λ}}^{-1}h_{11}{\text{Λ}}^{-1}={\tilde{\text{Λ}}}^{-1}$$,

we approximate the generalized inverse of ${\hat{F}}_{11}+\varepsilon {\hat{H}}_{11}$ as

(23) $${\left({\hat{F}}_{11}+\varepsilon {\hat{H}}_{11}\right)}^{†}\approx U\left[\begin{array}{cc} {\tilde{\text{Λ}}}^{-1}+{\tilde{\text{Λ}}}^{-1}\left(\varepsilon h_{12}\right)S^{†}\left(\varepsilon h_{12}^{⊤}\right){\tilde{\text{Λ}}}^{-1} & -{\tilde{\text{Λ}}}^{-1}\left(\varepsilon h_{12}\right)S^{†}\\ -S^{†}\left(\varepsilon h_{12}^{⊤}\right){\tilde{\text{Λ}}}^{-1} & S^{†} \end{array}\right]U^{⊤},$$

and the matrix S is approximated on the second-order of ε as follows:

(24) $$S\approx \varepsilon h_{22}-\varepsilon^{2}h_{12}^{⊤}{\text{Λ}}^{-1}h_{12}$$

and the generalized inverse of S is approximately expressed as

(25) $$S^{†}=\frac{1}{\varepsilon }{\left(h_{22}-\varepsilon h_{12}^{⊤}{\text{Λ}}^{-1}h_{12}\right)}^{†}\approx \frac{1}{\varepsilon }h_{22}^{†}+h_{22}^{†}\left(h_{12}^{⊤}{\text{Λ}}^{-1}h_{12}\right)h_{22}^{†}.$$

The loss function $L\left(\theta_{i};\tilde{\theta },\varepsilon \right)$ is rewritten as

(26) $$L\left(\theta_{i};\tilde{\theta },\varepsilon \right)\approx \left(K_{0}+K_{1}\varepsilon +K_{2}\varepsilon^{2}\right)\theta_{i}^{2}$$

Denoted as $\left[G_{1},G_{2}\right]=\left[{\hat{F}}_{21}U_{r},{\hat{F}}_{21}U_{k-r}\right]$, $\left[K_{1},K_{2}\right]=\left[{\hat{H}}_{21}U_{r},{\hat{H}}_{21}U_{k-r}\right]$, we note $G_{2}=0$ since the null space of ${\hat{F}}_{12}$ is the same as ${\hat{F}}_{11}$ (${\hat{F}}_{22}=s_{i}^{⊤}s_{i}$, ${\hat{F}}_{21}=s_{i}^{⊤}A$, ${\hat{F}}_{11}=A^{⊤}A$, where $s_{i}$ is the *i*^*th*^ column of sensitive matrix $s\left(\theta^{*}\right)=\left[\nabla_{\theta }h{\left(t_{1},\theta^{*}\right)}^{⊤};\nabla_{\theta }h{\left(t_{2},\theta^{*}\right)}^{⊤};\cdots ;\nabla_{\theta }h{\left(t_{N},\theta^{*}\right)}^{⊤}\right]$ and $A=\left[s_{1},s_{2},\cdots ,s_{i-1},s_{k},s_{i+1},\cdots ,s_{k-1}\right]$ (27)) so that the following equation is shown as:

(27) $$\begin{array}{l} {\hat{F}}_{22}+\varepsilon {\hat{H}}_{22}-\left({\hat{F}}_{21}+\varepsilon {\hat{H}}_{21}\right){\left({\hat{F}}_{11}+\varepsilon {\hat{H}}_{11}\right)}^{†}{\left({\hat{F}}_{21}+\varepsilon {\hat{H}}_{21}\right)}^{⊤}\\ ={\hat{F}}_{22}+\varepsilon {\hat{H}}_{22}-\left[\left(G_{1}+\varepsilon K_{1}\right)\left({\tilde{\text{Λ}}}^{-1}+\varepsilon^{2}{\tilde{\text{Λ}}}^{-1}h_{12}S^{†}h_{12}^{⊤}{\tilde{\text{Λ}}}^{-1}\right){\left(G_{1}+\varepsilon K_{1}\right)}^{⊤}\right.\\ \left.-\varepsilon^{2}K_{2}S^{†}h_{12}^{⊤}{\tilde{\text{Λ}}}^{-1}{\left(G_{1}+\varepsilon K_{1}\right)}^{⊤}-\varepsilon^{2}\left(G_{1}+\varepsilon K_{1}\right){\tilde{\text{Λ}}}^{-1}h_{12}S^{†}K_{2}^{⊤}+\varepsilon^{2}K_{2}S^{†}K_{2}^{⊤}\right] \end{array}$$

where $S^{†}$ and ${\tilde{\text{Λ}}}^{-1}$ are shown as

(28) $$\begin{array}{l} S^{†}\approx \frac{1}{\varepsilon }h_{22}^{†}+h_{22}^{†}\left(h_{12}^{⊤}{\text{Λ}}^{-1}h_{12}\right)h_{22}^{†},\\ {\tilde{\text{Λ}}}^{-1}={\text{Λ}}^{-1}-\varepsilon {\text{Λ}}^{-1}h_{11}{\text{Λ}}^{-1}+\varepsilon^{2}{\text{Λ}}^{-1}h_{11}{\text{Λ}}^{-1}h_{11}{\text{Λ}}^{-1} \end{array}$$

Now, we can quantify the coordinate practical identifiability for a specific parameter $\theta_{i}$ using the metric $\left(K_{i}\right)$ for a given ε-order under Theorem 2.

#### Theorem 2.

*Based on Eqs. 27 and 28, higher-order coordinate practical identifiability is quantified by the following three indices:*

1. *Zero-order* ε(O(1)),
(29) $$K_{0}={\hat{F}}_{22}-G_{1}{\text{Λ}}^{-1}G_{1}^{⊤}={\hat{F}}_{22}-{\hat{F}}_{21}F_{11}^{†}{\hat{F}}_{21}^{⊤}.$$
2. *First-order* ε(O(ε)),
(30) $$K_{1}={\hat{H}}_{22}-\left(K_{1}{\text{Λ}}^{-1}G_{1}^{⊤}+G_{1}{\text{Λ}}^{-1}K_{1}^{⊤}+K_{2}h_{22}^{†}K_{2}^{⊤}+G_{1}{\text{Λ}}^{-1}h_{12}h_{22}^{†}h_{12}^{⊤}{\text{Λ}}^{-1}G_{1}^{⊤}\right)\;\;+G_{1}{\text{Λ}}^{-1}h_{11}{\text{Λ}}^{-1}G_{1}^{⊤}+K_{2}h_{22}^{†}h_{12}^{⊤}{\text{Λ}}^{-1}G_{1}^{⊤}+G_{1}{\text{Λ}}^{-1}h_{12}h_{22}^{†}K_{2}^{⊤}$$
3. *Second-order* $\varepsilon^{2}\left(O\left(\varepsilon^{2}\right)\right)$,
(31) $$K_{2}=-\left\{K_{1}{\text{Λ}}^{-1}K_{1}^{⊤}-G_{1}{\text{Λ}}^{-1}h_{11}{\text{Λ}}^{-1}K_{1}^{⊤}-K_{1}{\text{Λ}}^{-1}h_{11}{\text{Λ}}^{-1}G_{1}^{⊤}+G_{1}{\text{Λ}}^{-1}h_{11}{\text{Λ}}^{-1}h_{11}{\text{Λ}}^{-1}G_{1}^{⊤}\right.\;\;+\left(G_{1}{\text{Λ}}^{-1}h_{12}-K_{2}\right)\left[h_{22}^{†}\left(h_{12}^{⊤}{\text{Λ}}^{-1}h_{12}\right)h_{22}^{†}\right]{\left(G_{1}{\text{Λ}}^{-1}h_{12}-K_{2}\right)}^{⊤}\;\;\left.+\left[\left(K_{1}{\text{Λ}}^{-1}-G_{1}{\text{Λ}}^{-1}h_{11}{\text{Λ}}^{-1}\right)h_{12}h_{22}^{†}{\left(G_{1}{\text{Λ}}^{-1}h_{12}-K_{2}\right)}^{⊤}\right)\right]\;\;\left.\left.+\left[\left(G_{1}{\text{Λ}}^{-1}h_{12}-K_{2}\right)h_{22}^{†}h_{12}^{⊤}{\left(K_{1}{\text{Λ}}^{-1}-G_{1}{\text{Λ}}^{-1}h_{11}{\text{Λ}}^{-1}\right)}^{⊤}\right)\right]\right\}$$

Moreover, we propose three corollaries on the three indices $\left(K_{i},i=0,1,2\right)$ as follows:

#### Corollary 2.

*The metric* $K_{0}$ *is equivalent with the metric* ${\left\|\left(I-AA^{†}s_{i}\right)\right\|}_{2}^{2}$ *where* $s_{i}$ *is the i^th^ column of sensitive matrix* $s\left(\theta^{*}\right)=\left[\nabla_{\theta }h{\left(t_{1},\theta^{*}\right)}^{⊤};\nabla_{\theta }h{\left(t_{2},\theta^{*}\right)}^{⊤};\cdots ;\nabla_{\theta }h{\left(t_{N},\theta^{*}\right)}^{⊤}\right]$ *and* $A=\left[s_{1},s_{2},\cdots ,s_{i-1},s_{k},s_{i+1},\cdots ,s_{k-1}\right]$ (27).

#### Corollary 3.

*When matrix* H *is a postive semi-definite matrix, the index* $K_{1}\geq 0$.

#### Corollary 4.

*When matrix* H *is a postive semi-definite matrix and* $K_{1}=0$, *the index* $K_{2}$ *is zero* $\left(K_{2}=0\right)$.

Details of these proofs are shown in the corresponding section “Proof of Corollary 2”, “Proof of Corollary 3”, and “Proof of Corollary 4” in Supplementary Materials, respectively.

### UQ for Practical Non-identifiable Parameters

Our previous work established an UQ methodology grounded in practical identifiability to evaluate how variations in non-identifiable parameters influence model uncertainty (27). For those aligned with the non-identifiable eigenvectors $U_{k-r}^{T}$, we perform a perturbation vector as $\varepsilon_{k-r}∼N\left(0,{\text{Σ}}_{k-r}\right)\left(U_{k-r}^{T}\text{Δ}\theta =\varepsilon_{k-r}\right)$. The model parameters are adjusted by:

(32) $$\text{Δ}\theta =\hat{\theta }-\theta^{*}=U_{k-r}\varepsilon_{k-r}$$

The observable variable $h\left(\varphi \left(t,\theta^{*}\right)\right.$ is linearly approached as

(33) $$h(t,\hat{\theta })=h\left(t,\theta^{*}\right)+\nabla_{\theta }h\left(t,\theta^{*}\right)\text{Δ}\theta$$

Based on law of propagation of uncertainty, the estimation of uncertainty on the observable variable such as $h_{l}(\varphi (t,\hat{\theta }))(l=1,2,\ldots ,L)$, ∀t>0 is written as:

(34) $$\text{Var}\left(h_{l}\left(t,\theta^{*}\right)\right)=\nabla_{\theta }h_{l}\left(t,\theta^{*}\right)\text{CoV}\left(\theta^{*}\right){\left(\nabla_{\theta }h_{l}\left(t,\theta^{*}\right)\right)}^{⊤}$$

where the variance of parameter $\hat{\theta }$ is obtain as $\text{CoV}(\hat{\theta })=U_{k-r}{\text{Σ}}_{k-r}U_{k-r}^{T}$. Through the linear approximation, the variance of the state variable is calculated using the error propagation formula, which can then be used to construct the confidence interval for the state variable. Assuming each component of observable variable $h_{l}(t,\hat{\theta })(l=1,2,\ldots ,L)$ approximately follows a normal distribution, its 100(1−α)% confidence interval follows:

(35) $$h_{l}(t,\hat{\theta })\in \left[h_{l}\left(t,\theta^{*}\right)-z_{\alpha /2}\sqrt{\text{Var}\left(h_{l}\left(t,\theta^{*}\right)\right)},h_{l}\left(t,\theta^{*}\right)+z_{\alpha /2}\sqrt{\text{Var}\left(h_{l}\left(t,\theta^{*}\right)\right)}\right]$$

where $z_{\alpha /2}$ is the critical value of the standard normal distribution. Now, we extend the uncertainty quantification to first-order ε non-identifiable parameter in Theorem 3 as follows:

#### Theorem 3.

*Define the uncertainty for the zero-order and first-order* ε *as* $Var_{0}\left(h_{l}\right)$ *and* $Var_{1}\left(h_{l}\right)$, *and assume the perturbation* $\text{Σ}=\sigma^{2}I$. *Following the UQ method, we have* $Var_{0}\left(h_{l}\right)\geq Var_{1}\left(h_{l}\right)$.

Then, we compute the uncertainty of ε-order for the loss function $l(h;\hat{h})$. The oss function $l(h;\hat{h})$ under the perturbation of data is rewritten as

(36) $$l\left(h\left(\theta_{\delta }\right);\hat{h}-\delta \right)\approx \frac{m\varepsilon^{2}}{2}+\varepsilon \left(\sum_{l=1}^{L}\nabla_{\theta }h_{l}\left(\theta^{*}\right)\right)⊤\text{Δ}\theta +\frac{1}{2}\text{Δ}\theta^{⊤}(F+\varepsilon H)\text{Δ}\theta$$

and the matrix F+εH can be expanded as

(37) $$F+\varepsilon H\approx \hat{U}\left[\begin{array}{ccc} {\text{Σ}}_{r_{0}}+B_{0} & & \\ & \varepsilon \left({\text{Σ}}_{r_{1}}+B_{1}\right) & \\ & & \varepsilon^{2}S_{2} \end{array}\right]{\hat{U}}^{⊤}$$

where the matrices $B_{0}$, $B_{1}$, and $S_{2}$ are shown as follows:

(38) $$\begin{array}{c} B_{0}=\varepsilon U_{r_{0}}^{(0)⊤}HU_{r_{0}}^{\left(0\right)},B_{1}=-\varepsilon U_{r_{1}}^{(1)⊤}H_{1}U_{r_{1}}^{\left(1\right)},S_{2}=-U_{k-\sum_{i=0}^{1}r_{i}}^{(1)⊤}H_{1}U_{k-\sum_{i=0}^{1}r_{i}}^{\left(1\right)};\\ H_{1}=U_{k-r_{0}}^{(0)⊤}H_{0}U_{r_{0}}^{\left(0\right)}{\text{Σ}}_{r_{0}}^{-1}U_{r_{0}}^{(0)⊤}H_{0}U_{k-r_{0}}^{\left(0\right)},Z_{1}=\varepsilon U_{k-r_{0}}^{(0)⊤}H_{0}U_{r_{0}}^{\left(0\right)}{\left({\text{Σ}}_{r_{0}}+\varepsilon U_{r_{0}}^{(0)⊤}H_{0}U_{r_{0}}^{\left(0\right)}\right)}^{-1};\\ \hat{U}=\left[U_{r_{0}}^{\left(0\right)}+U_{k-r_{0}}^{\left(0\right)}Z_{1},U_{k-r_{0}}^{\left(0\right)}U_{r_{1}}^{\left(1\right)},U_{k-r_{0}}^{\left(0\right)}U_{k-\sum_{i=0}^{1}r_{i}}^{\left(1\right)}\right]. \end{array}$$

Then, we decompose the perturbation Δθ using the projection matrix $\hat{U}$ as

(39) $$\text{Δ}\theta =\hat{U}\left[\begin{array}{c} 0\\ \eta_{r_{1}}\\ \eta_{k-\sum_{i=0}^{1}r_{i}} \end{array}\right]=U_{k-r_{0}}^{\left(0\right)}U_{r_{1}}^{\left(1\right)}\eta_{r_{1}}+U_{k-r_{0}}^{\left(0\right)}U_{k-\sum_{i=0}^{1}r_{i}}^{\left(1\right)}\eta_{k-\sum_{0}^{1}r_{i}}$$

where $\eta_{r_{1}}$ corresponds to the perturbations that are unidentifiable in the zeroth order but are actually identifiable in the first order and $\eta_{r_{1}}$ is the first-order perturbation which direction is still unidentifiable. The uncertainty of L-2 loss is dominated by the quadratic term denoted as

(40) $$l_{unc}=\frac{1}{2}\text{Δ}\theta^{⊤}(F+\varepsilon H)\text{Δ}\theta .$$

Substituting the $\eta_{r_{1}}$, the uncertainty of zero-order $\varepsilon \left(l_{unc}^{\left(0\right)}\right)$ is computed as follows:

(41) $$\begin{array}{l} l_{unc}^{\left(0\right)}=\frac{1}{2}{\left(U_{k-r_{0}}^{\left(0\right)}U_{r_{1}}^{\left(1\right)}\eta_{r_{1}}\right)}^{⊤}(F+\varepsilon H)\left(U_{k-r_{0}}^{\left(0\right)}U_{r_{1}}^{\left(1\right)}\eta_{r_{1}}\right)\\ \;=\frac{1}{2}\varepsilon \cdot \eta_{r_{1}}^{⊤}\left({\text{Σ}}_{r_{1}}+B_{1}\right)\eta_{r_{1}}. \end{array}$$

Substituting the $\eta_{k-\sum_{i=0}^{1}r_{i}}$, the uncertainty of first-order $\varepsilon \;\left(l_{unc}^{\left(1\right)}\right)$ is computed as:

(42) $$l_{unc}^{\left(1\right)}=\frac{1}{2}{\left(U_{k-r_{0}}^{\left(0\right)}U_{k-\sum_{0}^{1}r_{i}}^{\left(1\right)}\eta_{k-\sum_{0}^{1}r_{i}}\right)}^{⊤}(F+\varepsilon H)\left(U_{k-r_{0}}^{\left(0\right)}U_{k-\sum_{0}^{1}r_{i}}^{\left(1\right)}\eta_{k-\sum_{0}^{1}r_{i}}\right)\;\;=\frac{1}{2}\varepsilon^{2}\cdot \eta_{k-{\sum_{i=0}^{i=1}r_{i}}_{i}}^{⊤}S_{2}\eta_{k-{\sum_{i=0}^{i=1}r_{i}}_{i}}$$

Assuming the homogeneous Gaussian distribution $\eta_{k-r_{0}}∼N\left(0,\sigma^{2}I_{k-r_{0}}\right)$, the expected loss of zero-order is computed using the formula $E\left(\eta^{⊤}M\eta \right)=\text{Tr}(M\text{CoV}(\eta ))$ as follows,

(43) $$E\left(l_{unc}^{\left(0\right)}\right)=\frac{1}{2}\sigma^{2}\varepsilon \cdot \text{Tr}\left({\text{Σ}}_{r_{1}}+B_{1}\right),$$

and variance of the loss

(44) $$\text{Var}\left(l_{unc}^{\left(0\right)}\right)=\frac{1}{2}\sigma^{4}\varepsilon^{2}\cdot \text{Tr}\left({\left({\text{Σ}}_{r_{1}}+B_{1}\right)}^{2}\right).$$

The expected loss of first-order ε is computed as

(45) $$E\left(l_{unc}^{\left(1\right)}\right)=\frac{1}{2}\sigma^{2}\varepsilon^{2}\cdot \text{Tr}\left(S_{2}\right),$$

and variance of the loss

(46) $$\text{Var}\left(l_{unc}^{\left(1\right)}\right)=\frac{1}{2}\sigma^{4}\varepsilon^{4}\cdot \text{Tr}\left(S_{2}^{2}\right).$$

**Algorithm 1:** Higher-Order Practical Identifiability and Uncertainty Quantification Analysis

**Input:** model φ(t,θ), observable variable h(⋅), empirical data ${\left\{\left(t_{i},{\hat{h}}_{i}\right)\right\}}_{i=1}^{N}$, perturbation amplitude σ	
**Output:** eigen-coordinate $\hat{U}$, metrics $K_{0}$ and $K_{1}$, variances $\text{Va}{\text{r}}_{0}$ and $\text{Va}{\text{r}}_{1}$	
1:	Compute the optimal parameter $\theta^{*}$ via least-squares estimation (Eq. 3)
2:	Compute the FIM F and perturbation Hessian matrix H by Eq. 9
3:	▷ *Higher-Order Practical Identifiability Analysis*
4:	Decompose matrix F as
$F=\left[\begin{array}{ll} U_{r_{0}}^{\left(0\right)} & U_{k-r_{0}}^{\left(0\right)} \end{array}\right]\left[\begin{array}{cc} {\text{Σ}}_{r_{0}} & 0\\ 0 & 0 \end{array}\right]{\left[\begin{array}{ll} U_{r_{0}}^{\left(0\right)} & U_{k-r_{0}}^{\left(0\right)} \end{array}\right]}^{⊤}.$	
	Zero-order identifiable eigen-coordinate $U_{r_{0}}^{(0)⊤}\theta$, non-identifiable eigen-coordinate $U_{k-r_{0}}^{(0)⊤}\theta$
5:	Decompose matrix $F_{1}=U_{k-r_{0}}^{(0)⊤}HU_{k-r_{0}}^{\left(0\right)}$ as
$F_{1}=\left[\begin{array}{ll} U_{r_{1}}^{\left(1\right)} & U_{k-r_{0}-r_{1}}^{\left(1\right)} \end{array}\right]\left[\begin{array}{cc} {\text{Σ}}_{r_{1}} & 0\\ 0 & 0 \end{array}\right]{\left[\begin{array}{ll} U_{r_{1}}^{\left(1\right)} & U_{k-r_{0}-r_{1}}^{\left(1\right)} \end{array}\right]}^{⊤}.$	
	First-order identifiable eigen-coordinate $U_{r_{1}}^{(1)⊤}U_{k-r_{0}}^{(0)⊤}\theta$, non-identifiable eigen-coordinate $U_{k-r_{1}-r_{0}}^{(1)⊤}U_{k-r_{0}}^{(0)⊤}\theta$
6:	▷ *Hierarchical Coordinate Identifiability Analysis*
7:	**for** i=1 **to** K **do**
8:	Compute metric $K_{0}$ by Eq. 29 and $K_{1}$ by Eq. 30
9:	**end for**
10:	▷ *Higher-Order Uncertainty Quantification (UQ) Analysis*
11:	**for** l=1 **to** L **do**
12:	Compute the zero-order uncertainty:
$\text{Va}{\text{r}}_{0}\left(h_{l}\left(t,\theta^{*}\right)\right)=\sigma^{2}\nabla_{\theta }h_{l}\left(t,\theta^{*}\right)\left(U_{k-r_{0}}^{\left(0\right)}U_{k-r_{0}}^{(0)⊤}\right){\left(\nabla_{\theta }h_{l}\left(t,\theta^{*}\right)\right)}^{⊤}$	
13:	Compute the first-order uncertainty:
$\text{Va}{\text{r}}_{1}\left(h_{l}\left(t,\theta^{*}\right)\right)=\sigma^{2}\nabla_{\theta }h_{l}\left(t,\theta^{*}\right)\left(U_{k-r_{0}}^{\left(0\right)}U_{k-\text{Σ}r_{i}}^{\left(1\right)}\right){\left(U_{k-r_{0}}^{\left(0\right)}U_{k-\text{Σ}r_{i}}^{\left(1\right)}\right)}^{⊤}{\left(\nabla_{\theta }h_{l}\left(t,\theta^{*}\right)\right)}^{⊤}$	
14:	**end for**
15:	**return** $\hat{U}$, $K_{0}$, $K_{1}$, $\text{Va}{\text{r}}_{0}$, $\text{Va}{\text{r}}_{1}$

## Supplementary Material

Supplement 1

## Data Availability

The data used in this study were obtained from the publicly available dataset in (38). Each data point consists of two values: the first indicates the relative time in days, and the second represents the viral concentration in thousands of HIV-1 RNA copies per milliliter (ml^−1^). Data used in the preparation of this article were obtained from the ADNI database (http://adni.loni.usc.edu/). ADNI was launched in 2003 as a public-private partnership, led by Principal Investigator Michael W. Weiner, MD. Its primary goal is to determine whether serial magnetic resonance imaging (MRI), positron emission tomography (PET), other biological markers, and clinical and neuropsychological assessments can be combined to track the progression of mild cognitive impairment (MCI) and early Alzheimer’s disease (AD). Based on our group preivous work (40) on the data preprocessing, we accessed multimodal neuroimaging and biomarker data from the ADNI database under an approved data use application. *Aβ*-PET standardized uptake value ratios (SUVRs) was obtained from the datasets titled *“UC Berkeley – Amyloid PET 6mm Res analysis [ADNI1, GO, 2, 3, 4]”*. Regional SUVRs were computed by dividing the standardized uptake values (SUVs) in target regions by the SUV of the whole cerebellum, chosen as the reference region due to its minimal nonspecific binding and stable tracer uptake across subjects. Source codes and data have been deposited on the GitHub repository (https://github.com/WilliamMoriaty/Scaling-Law-Practical-Identifiability).

## References

[1] La Manno G., , RNA velocity of single cells. Nature 560 (7719), 494–498 (2018). 30089906 10.1038/s41586-018-0414-6 PMC6130801

[2] Qiu X., , Mapping transcriptomic vector fields of single cells. Cell 185 (4), 690–711 (2022). 35108499 10.1016/j.cell.2021.12.045 PMC9332140

[3] Bocci F., Zhou P., Nie Q., spliceJAC: transition genes and state-specific gene regulation from single-cell transcriptome data. Molecular Systems Biology 18 (11), MSB202211176 (2022). 10.15252/msb.202211176 PMC9627675 36321549

[4] Su Z., Tong Y., Wei G.-W., Hodge decomposition of single-cell RNA velocity. Journal of chemical information and modeling 64 (8), 3558–3568 (2024). 38572676 10.1021/acs.jcim.4c00132 PMC11035094

[5] Weiler P., Lange M., Klein M., Pe’er D., Theis F., CellRank 2: unified fate mapping in multiview single-cell data. Nature Methods 21 (7), 1196–1205 (2024). 38871986 10.1038/s41592-024-02303-9 PMC11239496

[6] Yan L., Zhang D., Sun X., Decoding cell state transitions driven by dynamic cell–cell communication in spatial transcriptomics. Nature Computational Science pp. 1–15 (2026). 41491113 10.1038/s43588-025-00934-2

[7] Anderson H. G., , Global stability and parameter analysis reinforce therapeutic targets of PD-L1-PD-1 and MDSCs for glioblastoma. Journal of Mathematical Biology 88 (1), 10 (2024). 10.1007/s00285-023-02027-y PMC10724342 38099947

[8] Gatenbee C. D., , Immunosuppressive niche engineering at the onset of human colorectal cancer. Nature Communications 13 (1), 1798 (2022). 10.1038/s41467-022-29027-8 PMC8979971 35379804

[9] Li X., , Infiltration of CD8+ T cells into tumor cell clusters in triple-negative breast cancer. Proceedings of the National Academy of Sciences 116 (9), 3678–3687 (2019). 10.1073/pnas.1817652116 PMC6397588 30733298

[10] Liu Z., Zhang J., Hong L., Nie Q., Sun X., Multiscale mathematical model-informed reinforcement learning optimizes combination treatment scheduling in glioblastoma evolution. Science Advances 11 (32), eadv3316 (2025). 10.1126/sciadv.adv3316 PMC13155510 40779623

[11] Liao K.-L., Watt K. D., Mathematical Modeling and Analysis of CD200–CD200R in Cancer Treatment. Bulletin of Mathematical Biology 84 (8), 82 (2022). 35792958 10.1007/s11538-022-01039-x

[12] Petrella J. R., , Personalized Computational Causal Modeling of the Alzheimer Disease Biomarker Cascade. The journal of prevention of Alzheimer’s disease 11 (2), 435–444 (2024). 10.14283/jpad.2023.134 PMC11082854 38374750

[13] Hao W., Lenhart S., Petrella J. R., Optimal anti-amyloid-beta therapy for Alzheimer’s disease via a personalized mathematical model. PLoS computational biology 18 (9), e1010481 (2022). 10.1371/journal.pcbi.1010481 PMC9477429 36054214

[14] Rabiei K., , Data-driven modeling of amyloid-β targeted antibodies for Alzheimer’s disease. NPJ systems biology and applications 11 (1), 134 (2025). 41271757 10.1038/s41540-025-00610-1 PMC12638957

[15] Zheng H., , Data-driven causal model discovery and personalized prediction in Alzheimer’s disease. NPJ digital medicine 5 (1), 137 (2022). 36076010 10.1038/s41746-022-00632-7 PMC9458727

[16] Raue A., , Structural and practical identifiability analysis of partially observed dynamical models by exploiting the profile likelihood. Bioinformatics 25 (15), 1923–1929 (2009). 19505944 10.1093/bioinformatics/btp358

[17] Mitra E. D., Hlavacek W. S., Parameter estimation and uncertainty quantification for systems biology models. Current opinion in systems biology 18, 9–18 (2019). 32719822 10.1016/j.coisb.2019.10.006 PMC7384601

[18] Miao H., Xia X., Perelson A. S., Wu H., On identifiability of nonlinear ODE models and applications in viral dynamics. SIAM review 53 (1), 3–39 (2011). 21785515 10.1137/090757009 PMC3140286

[19] Portela A., Banga J. R., Matabuena M., Conformal prediction for uncertainty quantification in dynamic biological systems. PLOS Computational Biology 21 (5), e1013098 (2025). 10.1371/journal.pcbi.1013098 PMC12091895 40354480

[20] Rockne R. C., , The future of mathematical oncology in the age of AI. npj Systems Biology and Applications (2026). 10.1038/s41540-026-00656-9 PMC12890909 41588010

[21] Liu Y., Suh K., Maini P. K., Cohen D. J., Baker R. E., Parameter identifiability and model selection for partial differential equation models of cell invasion. Journal of the Royal Society Interface 21 (212) (2024). 10.1098/rsif.2023.0607 PMC10914513 38442862

[22] Bergman D. R., Jackson T., Jain H. V., Norton K.-A., An efficient and flexible framework for inferring global sensitivity of agent-based model parameters. PLoS computational biology 21 (9), e1013427 (2025). 10.1371/journal.pcbi.1013427 PMC12435692 40920814

[23] Gallo L., Frasca M., Latora V., Russo G., Lack of practical identifiability may hamper reliable predictions in COVID-19 epidemic models. Science advances 8 (3), eabg5234 (2022). 10.1126/sciadv.abg5234 PMC8769547 35044820

[24] Ciocanel M.-V., , Parameter identifiability in PDE models of fluorescence recovery after photobleaching. Bulletin of Mathematical Biology 86 (4), 36 (2024). 38430382 10.1007/s11538-024-01266-4

[25] Eisenberg M. C., Jain H. V., A confidence building exercise in data and identifiability: Modeling cancer chemotherapy as a case study. Journal of theoretical biology 431, 63–78 (2017). 28733187 10.1016/j.jtbi.2017.07.018 PMC6007023

[26] Simpson M. J., Baker R. E., Parameter Identifiability, Parameter Estimation, and Model Prediction for Differential Equation Models. SIAM Review 68 (1), 153–171 (2026).

[27] A Systematic Computational Framework for Practical Identifiability Analysis in Mathematical Models Arising from Biology. Advanced Science 12 (35), e04346 (2025). 10.1002/advs.202504346 PMC12463036 40693285

[28] Rodriguez K., , Concentration-dependent transcriptional switching through a collective action of cis-elements. Science advances 8 (31), eabo6157 (2022). 10.1126/sciadv.abo6157 PMC9365274 35947668

[29] Nguyen V. K., Binder S. C., Boianelli A., Meyer-Hermann M., Hernandez-Vargas E. A., Ebola virus infection modeling and identifiability problems. Frontiers in microbiology 6, 257 (2015). 25914675 10.3389/fmicb.2015.00257 PMC4391033

[30] Sharomi O., Gumel A. B., Mathematical Analysis of HIV Treatment Model with Variable Viral Load and Infection Stages., in Modeling Paradigms and Analysis of Disease Trasmission Models (2010), pp. 209–247.

[31] Borisov I., Metelkin E., Confidence intervals by constrained optimization—An algorithm and software package for practical identifiability analysis in systems biology. PLOS Computational Biology 16 (12), e1008495 (2020). 10.1371/journal.pcbi.1008495 PMC7785248 33347435

[32] Mirzaei N. M., Hao W., Shahriyari L., Investigating the spatial interaction of immune cells in colon cancer. Iscience 26 (5) (2023). 10.1016/j.isci.2023.106596 PMC10165418 37168560

[33] Kreutz C., An easy and efficient approach for testing identifiability. Bioinformatics 34 (11), 1913–1921 (2018). 29365095 10.1093/bioinformatics/bty035

[34] Wieland F.-G., Hauber A. L., Rosenblatt M., Tönsing C., Timmer J., On structural and practical identifiability. Current Opinion in Systems Biology 25, 60–69 (2021).

[35] Monsalve-Bravo G. M., , Analysis of sloppiness in model simulations: Unveiling parameter uncertainty when mathematical models are fitted to data. Science advances 8 (38), eabm5952 (2022). 10.1126/sciadv.abm5952 PMC9491719 36129974

[36] Raue A., Kreutz C., Theis F. J., Timmer J., Joining forces of Bayesian and frequentist methodology: a study for inference in the presence of non-identifiability. Philosophical Transactions of the Royal Society A: Mathematical, Physical and Engineering Sciences 371 (1984), 20110544 (2013). 10.1098/rsta.2011.0544 23277602

[37] Perelson A. S., Modelling viral and immune system dynamics. Nature reviews immunology 2 (1), 28–36 (2002). 10.1038/nri700 11905835

[38] Stafford M. A., , Modeling plasma virus concentration during primary HIV infection. Journal of theoretical biology 203 (3), 285–301 (2000). 10716909 10.1006/jtbi.2000.1076

[39] Golub G. H., Van Loan C. F., Matrix computations (JHU press) (2013).

[40] Wang J., , Learning patient-specific spatial biomarker dynamics via operator learning for Alzheimer’s disease progression. ArXiv pp. arXiv–2507 (2025). 10.1038/s41540-026-00719-x PMC13346838 42082519

[41] Li C., Mao Y., Liu X., Hao W., Data-driven spatiotemporal modeling reveals personalized trajectories of cortical atrophy in Alzheimer’s disease. arXiv preprint arXiv:2511.08847 (2025).

