# Supplementary material for: Unveiling Scaling Laws of Parameter Identifiability and Uncertainty Quantification in Data-Driven Biological Modeling
Source: ArXiv. 2026 Feb 24:arXiv:2602.20495v1. Preprint. [Version 1] (PMC12970388)
Supplement: Supplement 1 [file NIHPP2602.20495v1-supplement-1.pdf]

Supplementary materials

Proofs of Theorem 1-3 and Corollary 1-4

Supplementary Text

Figures S1

Tables S1 to S8

Supplementary Materials for Unveiling Scaling Laws of Parameter Identifiability and Uncertainty Quantification in Data-Driven Biological Modeling

Shun Wang, Wenrui Hao*

Email: wxh64@psu.edu

This PDF file includes:

Proofs of Theorem 1-3 and Corollary 1-4

Supplementary Text

Figures S1

Tables S1 to S8

Proofs of Theorems and Corollaries

Proof of Theorem 1

Proof. Perform the eigenvalue decomposition to the symmetric matrix F :

$$F = \begin{bmatrix} U_r & U_{k-r} \end{bmatrix} \begin{bmatrix} \Sigma_r & 0 \\ 0 & 0 \end{bmatrix} \begin{bmatrix} U_r & U_{k-r} \end{bmatrix}^\top . \tag{S1}$$

Now consider:

$$F + \varepsilon H = \begin{bmatrix} U_r & U_{k-r} \end{bmatrix} \begin{bmatrix} \Sigma_r + \varepsilon U_r^\top H U_r & \varepsilon U_r^\top H U_{k-r} \\ \varepsilon U_{k-r}^\top H U_r & \varepsilon U_{k-r}^\top H U_{k-r} \end{bmatrix} \begin{bmatrix} U_r & U_{k-r} \end{bmatrix}^\top . \tag{S2}$$

Let us define:

$$Q := \begin{bmatrix} \Sigma_r + \varepsilon U_r^\top H U_r & \varepsilon U_r^\top H U_{k-r} \\ \varepsilon U_{k-r}^\top H U_r & \varepsilon U_{k-r}^\top H U_{k-r} \end{bmatrix} . \tag{S3}$$

Following Definition 2, the Schur complement matrix of $(\Sigma_{r_0} + \varepsilon U_{r_0}^{(0)\top} H U_{r_0}^{(0)})$ is obtained as:

$$S_1 = \varepsilon \Phi_1 = \varepsilon \big(U_{k-r_0}^{(0)\top} H_0 U_{k-r_0}^{(0)} - \varepsilon U_{k-r_0}^{(0)\top} H_0 U_{r_0}^{(0)} (\Sigma_{r_0} + \varepsilon U_{r_0}^{(0)\top} H_0 U_{r_0}^{(0)})^{-1} U_{r_0}^{(0)\top} H_0 U_{k-r_0}^{(0)} \big). \tag{S4}$$

where $H_0 = H$, $U_{r_0}^{(0)} = U_r$, and $U_{k-r_0}^{(0)} = U_{k-r}$. The zero subspace of Q is determined by the zero eigenvectors of the following matrix Φ_1

$$\Phi_1 = U_{k-r_0}^{(0)\top} H_0 U_{k-r_0}^{(0)} - \varepsilon U_{k-r_0}^{(0)\top} H_0 U_{r_0}^{(0)} (\Sigma_{r_0} + \varepsilon U_{r_0}^{(0)\top} H_0 U_{r_0}^{(0)})^{-1} U_{r_0}^{(0)\top} H_0 U_{k-r_0}^{(0)} . \tag{S5}$$

By the $LDL^\top$ decomposition, we can rewrite the matrix Q_1 as:

$$Q_1 = L_1 \begin{bmatrix} \Sigma_{r_0} + \varepsilon U_{r_0}^{(0)\top} H_0 U_{r_0}^{(0)} & 0 \\ 0 & S_1 \end{bmatrix} L_1^\top, L_1 = \begin{bmatrix} I_{r_0} & 0 \\ Z_1 & I_{k-r_0} \end{bmatrix}, \tag{S6}$$

$$Z_1 = \varepsilon U_{k-r_0}^{(0)\top} H_0 U_{r_0}^{(0)} (\Sigma_{r_0} + \varepsilon U_{r_0}^{(0)\top} H_0 U_{r_0}^{(0)})^{-1}, \; B_0 = \varepsilon U_{r_0}^{(0)\top} H_0 U_{r_0}^{(0)}, \tag{S7}$$

$$F + \varepsilon H = U^{(0)} L_1 \begin{bmatrix} \Sigma_r + \varepsilon U_r^{(0)\top} H_0 U_r^{(0)} & 0 \\ 0 & S_1 \end{bmatrix} (U^{(0)} L_1)^\top, \tag{S8}$$

We expand the inverse term using the Neumann series:

$$(\Sigma_{r_0} + \varepsilon U_{r_0}^{(0)\top} H_0 U_{r_0})^{-1} = \left(I + \varepsilon \Sigma_{r_0}^{-1} U_{r_0}^{(0)\top} H_0 U_{r_0}^{(0)} \right)^{-1} \Sigma_{r_0}^{-1}, \tag{S9}$$

which is valid when the spectral radius $\rho(\varepsilon\Sigma_{r_0}^{-1}U_{r_0}^{(0)\top}H_0U_{r_0}^{(0)}) < 1$. Using Neumann series:

$$(\Sigma_{r_0} + \varepsilon U_{r_0}^{(0)\top}H_0U_{r_0}^{(0)})^{-1} = \sum_{n=0}^{\infty}(-1)^n(\varepsilon\Sigma_{r_0}^{-1}U_{r_0}^{(0)\top}H_0U_{r_0}^{(0)})^n\Sigma_{r_0}^{-1}. \tag{S10}$$

Thus, the matrix Φ_1 becomes:

$$\Phi_1 = U_{k-r_0}^{(0)\top}H_0U_{k-r_0}^{(0)} + U_{k-r_0}^{(0)\top}H_0U_{r_0}^{(0)}\left[\sum_{n=0}^{\infty}(-\varepsilon)^{n+1}(\Sigma_{r_0}^{-1}U_{r_0}^{(0)\top}H_0U_{r_0}^{(0)})^n\right]\Sigma_{r_0}^{-1}U_{r_0}^{(0)\top}H_0U_{k-r_0}^{(0)}. \tag{S11}$$

Expanding to the first-order term to Φ_1, that is the second-order term to $F + \varepsilon H$, we approximate the matrix Φ_1 as

$$\Phi_1 \approx U_{k-r_0}^{(0)\top}H_0U_{k-r_0}^{(0)} - \varepsilon U_{k-r_0}^{(0)\top}H_0U_{r_0}^{(0)}\Sigma_{r_0}^{-1}U_{r_0}^{(0)\top}H_0U_{k-r_0}^{(0)} \tag{S12}$$

Denoted as

$$\begin{aligned} F_1 &= U_{k-r_0}^{(0)\top}H_0U_{k-r_0}^{(0)}, \\ H_1 &= U_{k-r_0}^{(0)\top}H_0U_{r_0}^{(0)}\Sigma_{r_0}^{-1}U_{r_0}^{(0)\top}H_0U_{k-r_0}^{(0)}, \end{aligned} \tag{S13}$$

we rewrite the term $\Phi_1 \approx F_1 - \varepsilon H_1$. Then, we perform an eigenvalue decomposition on

$$F_1 = \begin{bmatrix} U_{r_1}^{(1)} & U_{k-\sum_{i=0}^{1}r_i}^{(1)} \end{bmatrix}\begin{bmatrix} \Sigma_{r_1} & 0 \\ 0 & 0 \end{bmatrix}\begin{bmatrix} U_{r_1}^{(1)\top} \\ U_{k-\sum_{i=0}^{1}r_i}^{(1)\top} \end{bmatrix}, \tag{S14}$$

and Φ_1 can also be rewritten as

$$\begin{aligned} \Phi_1 &\approx \begin{bmatrix} U_{r_1}^{(1)} & U_{k-\sum_{i=0}^{1}r_i}^{(1)} \end{bmatrix} Q_2 \begin{bmatrix} U_{r_1}^{(1)\top} \\ U_{k-\sum_{i=0}^{1}r_i}^{(1)\top} \end{bmatrix}, \\ Q_2 &= \begin{bmatrix} \Sigma_{r_1} - \varepsilon U_{r_1}^{(1)\top}H_1U_{r_1}^{(1)} & -\varepsilon U_{r_1}^{(1)\top}H_1U_{k-\sum_{i=0}^{1}r_i}^{(1)} \\ -\varepsilon U_{k-\sum_{i=0}^{1}r_i}^{(1)\top}H_1U_{r_1}^{(1)} & -\varepsilon U_{k-\sum_{i=0}^{1}r_i}^{(1)\top}H_1U_{k-\sum_{i=0}^{1}r_i}^{(1)\top} \end{bmatrix} \end{aligned} \tag{S15}$$

The Schu r complement of $\Sigma_{r_1} - \varepsilon U_{r_1}^{(1)\top}H_1U_{r_1}^{(1)}$ is written as

$$S_2 = -\varepsilon\Phi_2 = -\varepsilon\big(U_{k-\sum_{i=0}^{1}r_i}^{(1)\top}H_1U_{k-\sum_{i=0}^{1}r_i}^{(1)\top} + \varepsilon U_{k-\sum_{i=0}^{1}r_i}^{(1)\top}H_1U_{r_1}^{(1)}(\Sigma_{r_1} - \varepsilon U_{r_1}^{(1)\top}H_1U_{r_1}^{(1)})^{-1}U_{r_1}^{(1)\top}H_1U_{k-\sum_{i=0}^{1}r_i}^{(1)}\big) \tag{S16}$$

By the $LDL^\top$ decomposition, we can rewrite the matrix Q_2 as:

$$Q_2 = L_2\begin{bmatrix} \Sigma_{r_1} - \varepsilon U_{r_1}^{(1)\top}H_1U_{r_1}^{(1)} & 0 \\ 0 & S_2 \end{bmatrix}L_2^\top, L_2 = \begin{bmatrix} I_{r_1} & 0 \\ Z_2 & I_{k-\sum_{i=0}^{1}r_i} \end{bmatrix}. \tag{S17}$$

$$Z_2 = -\varepsilon U^{(1)\top}_{k-\sum_{i=0}^{1} r_i} H_1 U^{(1)}_{r_1} (\Sigma_{r_1} - \varepsilon U^{(1)\top}_{r_1} H_1 U^{(1)}_{r_1})^{-1},\ B_1 = -\varepsilon U^{(1)\top}_{r_1} H_1 U^{(1)}_{r_1}, \tag{S18}$$

$$F_1 - \varepsilon H_1 = U^{(1)} L_2 \begin{bmatrix} \Sigma_{r_1} - \varepsilon U^{(1)\top}_{r_1} H_1 U^{(1)}_{r_1} & 0 \\ 0 & S_2 \end{bmatrix} (U^{(1)} L_2)^\top \tag{S19}$$

And we use the linear approximation to the Φ_2 as

$$\Phi_2 \approx U^{(1)\top}_{k-\sum_{i=0}^{1} r_i} H_1 U^{(1)\top}_{k-\sum_{i=0}^{1} r_i} + \varepsilon U^{(1)\top}_{k-\sum_{i=0}^{1} r_i} H_1 U^{(1)}_{r_1} \Sigma_{r_1}^{-1} U^{(1)\top}_{r_1} H_1 U^{(1)}_{k-\sum_{i=0}^{1} r_i} \tag{S20}$$

Denoted as

$$\begin{aligned} F_2 &= U^{(1)\top}_{k-\sum_{i=0}^{1} r_i} H_1 U^{(1)}_{k-\sum_{i=0}^{1} r_i}, \\ H_2 &= U^{(1)\top}_{k-\sum_{i=0}^{1} r_i} H_1 U^{(1)}_{r_1} \Sigma_{r_1}^{-1} U^{(1)\top}_{r_1} H_1 U^{(1)}_{k-\sum_{i=0}^{1} r_i}, \end{aligned} \tag{S21}$$

we rewrite the $\Phi_2 \approx F_2 + \varepsilon H_2$. Then, we continue to perform the eigenvalue decomposition to F_2 as

$$F_2 = \begin{bmatrix} U^{(2)}_{r_2} & U^{(2)}_{k-\sum_{i=0}^{2} r_i} \end{bmatrix} \begin{bmatrix} \Sigma_{r_2} & 0 \\ 0 & 0 \end{bmatrix} \begin{bmatrix} U^{(2)\top}_{r_2} \\ U^{(2)\top}_{k-\sum_{i=0}^{2} r_i} \end{bmatrix}, \tag{S22}$$

and Φ_2 can also be rewritten as

$$\begin{aligned} \Phi_2 &\approx \begin{bmatrix} U^{(2)}_{r_2} & U^{(2)}_{k-\sum_{i=0}^{2} r_i} \end{bmatrix} Q_3 \begin{bmatrix} U^{(2)\top}_{r_2} \\ U^{(2)\top}_{k-\sum_{i=0}^{2} r_i} \end{bmatrix}, \\ Q_3 &= \begin{bmatrix} \Sigma_{r_2} + \varepsilon U^{(2)\top}_{r_2} H_2 U^{(2)}_{r_2} & \varepsilon U^{(2)\top}_{r_2} H_2 U^{(2)}_{k-\sum_{i=0}^{2} r_i} \\ \varepsilon U^{(2)\top}_{k-\sum_{i=0}^{2} r_i} H_2 U^{(2)}_{r_2} & \varepsilon U^{(2)\top}_{k-\sum_{i=0}^{2} r_i} H_2 U^{(2)\top}_{k-\sum_{i=0}^{2} r_i} \end{bmatrix} \end{aligned} \tag{S23}$$

The Schur complement of $\Sigma_{r_2} + \varepsilon U^{(2)\top}_{r_2} H_2 U^{(2)}_{r_2}$ is written as

$$S_3 = \varepsilon \Phi_3 = \varepsilon\big(U^{(2)\top}_{k-\sum_{i=0}^{2} r_i} H_2 U^{(2)\top}_{k-\sum_{i=0}^{2} r_i} + \varepsilon U^{(2)\top}_{k-\sum_{i=0}^{2} r_i} H_2 U^{(2)}_{r_2} (\Sigma_{r_2} + \varepsilon U^{(2)\top}_{r_2} H_2 U^{(2)}_{r_2})^{-1} U^{(2)\top}_{r_2} H_2 U^{(2)}_{k-\sum_{i=0}^{2} r_i}\big) \tag{S24}$$

By the $LDL^\top$ decomposition, we can rewrite the matrix Q_3 as:

$$Q_3 = L_3 \begin{bmatrix} \Sigma_{r_2} + \varepsilon U^{(2)\top}_{r_2} H_2 U^{(2)}_{r_2} & 0 \\ 0 & S_3 \end{bmatrix} L_3^\top, L_3 = \begin{bmatrix} I_{r_2} & 0 \\ Z_3 & I_{k-\sum_{i=0}^{2} r_i} \end{bmatrix}, \tag{S25}$$

$$Z_3 = \varepsilon U^{(2)\top}_{k-\sum_{i=0}^{2} r_i} H_2 U^{(2)}_{r_2} (\Sigma_{r_2} + \varepsilon U^{(2)\top}_{r_2} H_2 U^{(2)}_{r_2})^{-1},\ B_2 = \varepsilon U^{(2)\top}_{r_2} H_2 U^{(2)}_{r_2}, \tag{S26}$$

$$F_2 - \varepsilon H_2 = U^{(2)} L_3 \begin{bmatrix} \Sigma_{r_2} + \varepsilon U^{(2)\top}_{r_2} H_2 U^{(2)}_{r_2} & 0 \\ 0 & S_3 \end{bmatrix} (U^{(2)} L_3)^\top. \tag{S27}$$

So, we can compute the practical idenfitiability of order ε

(1) Zero-order ε ($O(1)$) for

$$F_0 = F \tag{S28}$$

, the non-identifiable parameter is

$$U_{k-r_0}^{(0)\top}\theta, \tag{S29}$$

and the practically identifiable parameter is

$$U_{r_0}^{(0)\top}\theta; \tag{S30}$$

(2) First-order ε ($O(\varepsilon)$) for

$$F_1 = U_{k-r_0}^{(0)\top} H_0 U_{k-r_0}^{(0)}, H_0 = H \tag{S31}$$

, the non-identifiable parameter is

$$U_{k-\sum_{i=0}^{1} r_i}^{(1)\top} U_{k-r_0}^{(0)\top}\theta, \tag{S32}$$

and the practically identifiable parameter is

$$[U_{r_0}^{(0)\top}\theta + Z_1^\top U_{k-r_0}^{(0)\top}\theta; U_{r_1}^{(1)\top} U_{k-r_0}^{(0)\top}\theta]; \tag{S33}$$

(3) Second-order ε ($O(\varepsilon^2)$) for

$$F_2 = U_{k-\sum_{i=0}^{1} r_i}^{(1)\top} H_1 U_{k-\sum_{i=0}^{1} r_i}^{(1)}, \quad H_1 = U_{k-r_0}^{(0)\top} H_0 U_{r_0}^{(0)} \Sigma_{r_0}^{-1} U_{r_0}^{(0)\top} H_0 U_{k-r_0}^{(0)}, \tag{S34}$$

the non-identifiable parameter is

$$U_{k-\sum_{i=0}^{2} r_i}^{(2)\top} U_{k-\sum_{i=0}^{1} r_i}^{(1)\top} U_{k-r_0}^{(0)\top}\theta, \tag{S35}$$

and the practically identifiable parameter is

$$[U_{r_0}^{(0)\top}\theta + Z_1^\top U_{k-r_0}^{(0)\top}\theta; U_{r_1}^{(1)\top} U_{k-r_0}^{(0)\top}\theta + Z_2^\top U_{k-r_o-r_1}^{(1)\top} U_{k-r_0}^{(0)\top}\theta; U_{r_2}^{(2)\top} U_{k-\sum_{i=0}^{1} r_i}^{(1)\top} U_{k-r_0}^{(0)\top}\theta]. \tag{S36}$$

Finally, the matrix $\hat{U}$, B_1,B_2, and B_3 are obtained as follows:

$$\hat{U} = [U_{r_0}^{(0)} + U_{k-r_0}^{(0)} Z_1, U_{k-r_0}^{(0)} U_{r_1}^{(1)} + U_{k-r_0}^{(0)} U_{k-r_o-r_1}^{(1)} Z_2, U_{k-r_0}^{(0)} U_{k-\sum_{i=0}^{1} r_i}^{(1)} U_{r_2}^{(2)}, U_{k-r_0}^{(0)} U_{k-\sum_{i=0}^{1} r_i}^{(1)} U_{k-\sum_{i=0}^{2} r_i}^{(2)}] \tag{S37}$$

□

Proof of Corollary 1

Proof. Since $H \succ 0$ and $H \in \text{null}(F) \setminus \{0\}$, and since U_{k-r} spans the null space of F, the block $\varepsilon U_{k-r}^\top H U_{k-r}$ is positive definite and hence invertible for $\varepsilon > 0$.

We compute the Schur complement of the bottom-right block in Eq. S3:

$$S = \Sigma_r + \varepsilon U_r^\top H U_r - \varepsilon^2 U_r^\top H U_{k-r} \left(U_{k-r}^\top H U_{k-r}\right)^{-1} U_{k-r}^\top H U_r. \tag{S38}$$

We now prove $S \succ 0$. For any $x \neq 0 \in \mathbb{R}^r$,

$$x^\top S x = x^\top \Sigma_r x + \varepsilon \left(x^\top U_r^\top H U_r x\right) - \varepsilon^3 x^\top U_r^\top H U_{k-r} \left(U_{k-r}^\top H U_{k-r}\right)^{-1} U_{k-r}^\top H U_r x. \tag{S39}$$

Using bounds: $x^\top \Sigma_r x \geq c_1 \|x\|^2$, where c_1 is the smallest eigenvalue of Σ_r, $x^\top U_r^\top H U_r x \leq \|H\| \cdot \|U_r x\|^2$, - The last term is bounded by $\frac{\varepsilon^3 \|U_{k-r}^\top H U_r\|^2 \|x\|^2}{c_2}$, where c_2 is the smallest eigenvalue of $U_{k-r}^\top H U_{k-r}$.

So there exists $\varepsilon_0 > 0$ such that for all $0 < \varepsilon < \varepsilon_0$,

$$x^\top S x \geq c_1 \|x\|^2 - \varepsilon \|H\| \|x\|^2 - \frac{\varepsilon^3 \|U_{k-r}^\top H U_r\|^2 \|x\|^2}{c_2} > 0. \tag{S40}$$

Hence $S \succ 0$, using Schur complement theory (*39*), $F + \varepsilon H$ is invertible. □

Proof of Corollary 2

Proof. The present metric $\mathcal{K}_0$ is the same as $\|(I - AA^\dagger s_i)\|_2^2$ is provided as follows:

$$\|(I - AA^\dagger s_i)\|_2^2 = s_i^\top s_i - s_i^\top (A^\dagger)^\top A^\top s_i - s_i^\top AA^\dagger s_i + s_i^\top (A^\dagger)^\top A^\top AA^\dagger s_i \tag{S41}$$

The generalized inverse is expressed using the EVD as:

$$A = U\Sigma V^\top,\ A^\dagger = V\Sigma^\dagger U^\top,\ A^\top = V\Sigma^\top U^\top,\ (A^\dagger)^\top = U(\Sigma^\dagger)^\top V^\top \tag{S42}$$

So, we have

$$\|(I - AA^\dagger s_i)\|_2^2 = s_i^\top s_i - s_i^\top U(\Sigma^\dagger)^\top \Sigma^\top U^\top s_i - s_i^\top U\Sigma\Sigma^\dagger U^\top s_i + s_i^\top U(\Sigma^\dagger)^\top \Sigma^\top \Sigma\Sigma^\dagger U^\top s_i \tag{S43}$$

$$\mathcal{K}_0 = s_i^\top s_i - s_i^\top U\Sigma V^\top V(\Sigma^\top\Sigma)^\dagger V^\top V\Sigma^\top U^\top s_i = s_i^\top s_i - s_i^\top U\Sigma(\Sigma^\top\Sigma)^\dagger \Sigma^\top U^\top s_i; \tag{S44}$$

$$(\Sigma^\dagger)^\top \Sigma^\top = \Sigma\Sigma^\dagger = (\Sigma^\dagger)^\top \Sigma^\top \Sigma\Sigma^\dagger = \Sigma(\Sigma^\top\Sigma)^\dagger \Sigma^\top = \begin{bmatrix} I_r & 0 \\ 0 & 0 \end{bmatrix} \tag{S45}$$

$$\mathcal{K}_0 = s_i^\top s_i - s_i^\top U \begin{bmatrix} I_r & 0 \\ 0 & 0 \end{bmatrix} U^\top s_i = \|(I - AA^\dagger s_i)\|_2^2. \tag{S46}$$

□

Proof of Corollary 3

Proof. First, the matrix $\hat{H}$ is also postive semi-definite, because of the postive semi-definite matrix H. Using the projection of matrix U, the matrix $\hat{H}$ is rewritten as

$$\tilde{H} = \begin{bmatrix} h_{11} & h_{12} & K_1^\top \\ h_{12} & h_{22} & K_2^\top \\ K_1 & K_2 & \hat{H}_{22} \end{bmatrix} \tag{S47}$$

$\tilde{H}$ is also postive semi-definite. Then, we consider the Schur Complement matrix of h_{22} as follows,

$$S = \begin{bmatrix} h_{11} & K_1^\top \\ K_1 & \hat{H}_{22} \end{bmatrix} - \begin{bmatrix} h_{12} \\ K_2 \end{bmatrix} h_{22}^\dagger \begin{bmatrix} h_{12}\top & K_2^\top \end{bmatrix} = \begin{bmatrix} h_{11} - h_{12}h_{22}^\dagger h_{12}^\top & K_1^\top - h_{12}h_{22}^\dagger K_2^\top \\ K_1 - K_2 h_{22}^\dagger h_{12}^\top & \hat{H}_{22} - K_2 h_{22} \dagger K_2^\top \end{bmatrix} \tag{S48}$$

(

$$\tilde{H} = \begin{bmatrix} h_{11} & h_{12}^\top & K_1^\top \\ h_{12} & h_{22} & K_2^\top \\ K_1 & K_2 & \hat{H}_{22} \end{bmatrix} \to \tilde{H} = \begin{bmatrix} h_{22} & h_{12} & K_2^\top \\ h_{12} & h_{11} & K_1^\top \\ K_2 & K_1 & \hat{H}_{22} \end{bmatrix} \text{(row-column permutation))} \tag{S49}$$

$$A = h_{22},\ B = [h_{12}\ K_2]^\top, C = \begin{bmatrix} h_{11} & K_1^\top \\ K_1 & \hat{H}_{22} \end{bmatrix}, S = C - BA^{-1}B^\top \tag{S50}$$

)

S is postive semi-definite based on Schur Complement Lemma. Now, we sort out the index $\mathcal{K}_1$ as

$$\begin{aligned} \mathcal{K}_1 =& \hat{H}_{22} - 2K_1\Lambda^{-1}G_1^\top + G_1\Lambda^{-1}h_{11}\Lambda^{-1}G_1^\top \\ & - (K_2 - G_1\Lambda^{-1}h_{12})h_{22}^\dagger(K_2 - G_1\Lambda^{-1}h_{12})^\top \end{aligned}$$

Define the vector $y = [-G_1\Lambda^{-1}, 1]$, and compute the quadratic form $y^\top Sy$ as

$$\begin{aligned} y^\top Sy =& [-G_1\Lambda^{-1}\ 1] \begin{bmatrix} h_{11} - h_{12}h_{22}^\dagger h_{12}^\top & K_1^\top - h_{12}h_{22}^\dagger K_2^\top \\ K_1 - K_2 h_{22}^\dagger h_{12}^\top & \hat{H}_{22} - K_2 h_{22} \dagger K_2^\top \end{bmatrix} [-G_1\Lambda^{-1}\ 1]\top \\ =& G_1\Lambda^{-1}(h_{11} - h_{12}h_{22}^\dagger h_{12}^\top)\Lambda^{-1}G_1^\top - 2G_1\Lambda^{-1}(K_1^\top - h_{12}h_{22}^\dagger K_2^\top) + \hat{H}_{22} - K_2 h_{22}^\dagger K_2^\top \\ =& \text{term } A + \text{term } B. \end{aligned} \tag{S51}$$

where

$$
\begin{aligned}
\text{term } A &= G_1\Lambda^{-1}h_{11}\Lambda^{-1}G_1^\top - 2G_1\Lambda^{-1}K_1^\top + \hat{H}_{22} \\
\text{term } B &= -(G_1\Lambda^{-1}h_{12}h_{22}^\dagger h_{12}^\top\Lambda^{-1}G_1^\top - 2G_1\Lambda^{-1}h_{12}h_{22}^\dagger K_2^\top + K_2h_{22}^\dagger K_2^\top) \\
&= -(K_2 - G_1\Lambda^{-1}h_{12})h_{22}^\dagger(K_2 - G_1\Lambda^{-1}h_{12})^\top
\end{aligned} \tag{S52}
$$

So, we obtain $\mathcal{K}_1 = y^\top S y$. Since S is positive semi-definite, we have $\mathcal{K}_1 \geq 0$. □

Proof of Corollary 4

Proof. When $\mathcal{K}_1 = 0$, the vector $y = [-G_1\Lambda^{-1}, 1]$ is at the null space of S and $Sy^\top = 0$. Now, we expand the $Sy^\top$ as follows,

$$
S\begin{bmatrix} -\Lambda^{-1}G_1^\top \\ 1 \end{bmatrix} = \begin{bmatrix} h_{11} - h_{12}h_{22}^\dagger h_{12}^\top & K_1^\top - h_{12}h_{22}^\dagger K_2^\top \\ K_1 - K_2h_{22}^\dagger h_{12}^\top & \hat{H}_{22} - K_2h_{22} \dagger K_2^\top \end{bmatrix}\begin{bmatrix} -\Lambda^{-1}G_1^\top \\ 1 \end{bmatrix} = \begin{bmatrix} 0 \\ 0 \end{bmatrix}. \tag{S53}
$$

Here, we obtain an important equation as follows:

$$
K_1 - G_1\Lambda^{-1}h_{11} = (K_2 - G_1\Lambda^{-1}h_{12})h_{22}^\dagger h_{12}^\top \tag{S54}
$$

Denote $X = K_1 - G_1\Lambda^{-1}h_{11},\ Y = (G_1\Lambda^{-1}h_{12} - K_2)h_{22}^\dagger h_{12}^\top$, and compute $\mathcal{K}_2$:

$$
\begin{aligned}
\text{term 1} =& K_1\Lambda^{-1}K_1^\top - G_1\Lambda^{-1}h_{11}\Lambda^{-1}K_1^\top - K_1\Lambda^{-1}h_{11}\Lambda^{-1}G_1^\top \\
& + G_1\Lambda^{-1}h_{11}\Lambda^{-1}h_{11}\Lambda^{-1}G_1^\top \\
=& (K_1 - G_1\Lambda^{-1}h_{11})\Lambda^{-1}(K_1 - G_1\Lambda^{-1}h_{11})^\top \\
=& X\Lambda^{-1}X^\top
\end{aligned} \tag{S55}
$$

$$
\begin{aligned}
\text{term 2} =& (G_1\Lambda^{-1}h_{12} - K_2)[h_{22}^\dagger(h_{12}^\top\Lambda^{-1}h_{12})h_{22}^\dagger](G_1\Lambda^{-1}h_{12} - K_2)^\top \\
=& ((G_1\Lambda^{-1}h_{12} - K_2)h_{22}^\dagger h_{12}^\top)\Lambda^{-1}((G_1\Lambda^{-1}h_{12} - K_2)h_{22}^\dagger h_{12}^\top)^\top \\
=& Y\Lambda^{-1}Y^\top
\end{aligned} \tag{S56}
$$

$$
\begin{aligned}
\text{term 3} =& (K_1\Lambda^{-1} - G_1\Lambda^{-1}h_{11}\Lambda^{-1})h_{12}h_{22}^\dagger(G_1\Lambda^{-1}h_{12} - K_2)^\top \\
=& X\Lambda^{-1}Y^\top
\end{aligned} \tag{S57}
$$

$$
\begin{aligned}
\text{term 4} =& (G_1\Lambda^{-1}h_{12} - K_2)h_{22}^\dagger h_{12}^\top(K_1\Lambda^{-1} - G_1\Lambda^{-1}h_{11}\Lambda^{-1})^\top \\
=& Y\Lambda^{-1}X^\top
\end{aligned} \tag{S58}
$$

$$
\mathcal{K}_2 = -\,(\text{term 1} + \text{term 2} + \text{term 3} + \text{term 4}) = -(X+Y)\Lambda^{-1}(X+Y)^\top \tag{S59}
$$

Because of $X + Y = 0$, the index $\mathcal{K}_2$ is computed as zero ($\mathcal{K}_2 = 0$). □

Proof of Theorem 3

Proof. First, the variance matrix of the zero-order and first-order ε are written as follows.

$$
\begin{aligned}
CoV_0 &= \sigma^2 U^{(0)}_{k-r_0} U^{(0)\top}_{k-r_o} \\
CoV_1 &= \sigma^2 (U^{(0)}_{k-r_0} U^{(1)}_{k-\sum_{i=0}^{1} r_i})(U^{(0)}_{k-r_0} U^{(1)}_{k-\sum_{i=0}^{1} r_i})^\top
\end{aligned} \tag{S60}
$$

Denote $\mathcal{S}_0 = \text{range}(U^{(0)}_{k-r_0})$ as zero-order unidentifiable subspace, and $\mathcal{S}_1 = \text{range}(U^{(0)}_{k-r_0} U^{(1)}_{k-\sum_{i=0}^{1} r_i})$ as first-order ε unidentifiable subspace. Specifically, $U^{(1)}_{k-\sum_{i=0}^{1} r_i}$ acts as a basis selection within the range of $U^{(0)}_{k-r_0}$:

$$
\text{range}(U^{(0)}_{k-r_0} U^{(1)}_{k-\sum_{i=0}^{1} r_i}) \subseteq \text{range}(U^{(0)}_{k-r_0}) \Longrightarrow \mathcal{S}_1 \subseteq \mathcal{S}_0. \tag{S61}
$$

Using the property of projection subspace, the projection of $(\nabla_\theta h_l)\top$ onto $\mathcal{S}_1$ is equivalent to projecting the already-projected vector $P_0(\nabla_\theta h_l)\top$ onto the smaller subspaces $\mathcal{S}_1$:

$$
P_{\mathcal{S}_1}(\nabla_\theta h_l)^\top = P_{\mathcal{S}_1}(P_{\mathcal{S}_0}(\nabla_\theta h_l)^\top) \tag{S62}
$$

By the properties of orthogonal projections (which are contractive operators), we have:

$$
\|P_{\mathcal{S}_1}(\nabla_\theta h_l)^\top\|_2^2 = \|P_{\mathcal{S}_1}(P_{\mathcal{S}_0}(\nabla_\theta h_l)^\top)\|_2^2 \leq \|P_{\mathcal{S}_0}(\nabla_\theta h_l)^\top\|_2^2 \tag{S63}
$$

Finally, we obtain the following inequality:

$$
Var_1(h_l) = \sigma^2 \|P_1(\nabla_\theta h_l)^\top\|_2^2 \leq \sigma^2 \|P_0(\nabla_\theta h_l)^\top\|_2^2 = Var_0(h_l) \tag{S64}
$$

□

Supplementary Text

Computing the FIM and H for ODE

We introduce how to obtain the gradient vector $\nabla_\theta h_l(t; \boldsymbol{\theta})$ and Hessian matrix $\nabla^2_\theta h_l(t; \boldsymbol{\theta})$ ($l = 1, 2, \cdots, L$) for ordinary differential equation (ODE). When we have an ODE as

$$
\frac{dx}{dt} = f(x; \boldsymbol{\theta}), \ x \in \mathbb{R}^n, \ f \in \mathbb{R}^n, \ \boldsymbol{\theta} \in \mathbb{R}^k, \tag{S65}
$$

we compute the gradient of $\boldsymbol{\theta}$ as

$$\frac{dy}{dt} = \frac{\partial}{\partial \theta}\frac{dx}{dt} = \frac{\partial}{\partial \theta} f(x;\theta),\ \ y = \nabla_{\boldsymbol{\theta}} x,\ \ y \in \mathbb{R}^{n\times k}. \tag{S66}$$

Then, we can write the ODE of each component of y as

$$\frac{d}{dt} y_{ij} = \frac{\partial}{\partial \theta_j} f_i(x;\theta) = \sum_{m=1}^{n} \frac{\partial f_i}{\partial x_m}\frac{\partial x_m}{\partial \theta_j} + \frac{\partial f_i}{\partial \theta_j} = \sum_{m=1}^{n} \frac{\partial f_i}{\partial x_m} y_{mj} + \frac{\partial f_i}{\partial \theta_j} \tag{S67}$$

Then, we futher calculate the Hessian tensor H as

$$\frac{d}{dt} z_{ijp} = \frac{\partial}{\partial \theta_p}\left(\sum_{m=1}^{n} \frac{\partial f_i}{\partial x_m} y_{mj} + \frac{\partial f_i}{\partial \theta_j}\right), z = \frac{\partial y}{\partial \theta},\ \ z_{ijp} = \frac{\partial y_{ij}}{\partial \theta_p},\ \ z \in \mathbb{R}^{n\times k\times k}. \tag{S68}$$

The Eq. S68 can be rewritten as four terms as follows

$$\begin{aligned}
&\text{Term 1}: \sum_{m=1}^{n} \frac{\partial f_i}{\partial x_m}\frac{\partial y_{mj}}{\partial \theta_p} = \sum_{m=1}^{n} \frac{\partial f_i}{\partial x_m} z_{mjp};\\
&\text{Term 2}: \sum_{m=1}^{n}\left(\sum_{l=1}^{n} \frac{\partial}{\partial x_l}\left(\frac{\partial f_i}{\partial x_m}\right)\frac{\partial x_l}{\partial \theta_p}\right) y_{mj} = \sum_{m,l}^{n} \frac{\partial^2 f_i}{\partial x_l \partial x_m} y_{lp} y_{mj};\\
&\text{Term 3}: \quad \sum_{m=1}^{n} \frac{\partial^2 f_i}{\partial x_m \partial \theta_p} y_{mj};\\
&\text{Term 4}: \quad \sum_{m=1}^{n} \frac{\partial}{\partial x_m}\left(\frac{\partial f_i}{\partial \theta_j}\right)\frac{\partial x_m}{\partial \theta_p} = \sum_{m=1}^{n} \frac{\partial^2 f_i}{\partial x_m \partial \theta_j} y_{mp};\\
&\text{Term 5}: \quad \frac{\partial^2 f_i}{\partial \theta_j \partial \theta_p}.
\end{aligned} \tag{S69}$$

And we sort our these terms as

$$\frac{d}{dt} z_{ijp} = \sum_{m=1}^{n} \frac{\partial f_i}{\partial x_m} z_{mjp} + \sum_{m,l}^{n} \frac{\partial^2 f_i}{\partial x_l \partial x_m} y_{lp} y_{mj} + \sum_{m=1}^{n} \frac{\partial^2 f_i}{\partial x_m \partial \theta_p} y_{mj} + \sum_{m=1}^{n} \frac{\partial^2 f_i}{\partial x_m \partial \theta_j} y_{mp} + \frac{\partial^2 f_i}{\partial \theta_j \partial \theta_p} \tag{S70}$$

When the observed variable is $h(x) \in \mathbb{R}^L$, the gradient matrix $\hat{y} \in \mathbb{R}^{L\times K}$ is computed as

$$\hat{y} = \frac{\partial h}{\partial \theta} = H(x)y,\ \ H(x) = \nabla_x h \tag{S71}$$

and Hessian tensor $\hat{z} \in \mathbb{R}^{L\times K\times K}$ is rewritten as

$$\hat{z} = \frac{\partial \hat{y}}{\partial \theta} = \frac{\partial H(x)}{\partial \theta} y + H(x)\frac{\partial y}{\partial \theta} = \frac{\partial H(x)}{\partial \theta} y + H(x) z \tag{S72}$$

$$\frac{\partial H(x)}{\partial \theta} = \frac{\partial H(x)}{\partial x}\frac{\partial x}{\partial \theta} = \mathcal{H}_{obs} \cdot y, \;\; \hat{z} = \mathcal{H}_{obs}[y, y] + H(x)z \tag{S73}$$

$$\hat{z}_{ijp} = \sum_{l=1}^{n}\sum_{r=1}^{n} \frac{\partial h_i}{\partial x_l \partial x_r} y_{lj} y_{rp} + \sum_{l=1}^{n} \frac{\partial h_i}{\partial x_l} z_{ljp} \;\; (i \in \{1, 2, \cdots, L\}, j, p \in \{1, 2, \cdots, K\}) \tag{S74}$$

With respect to the initial value of Eqs S67 and S70, we set zero matrix or tensor since the parameters are independent on the initial value.

HIV host-virus dynamics

The basic model of viral infection (*37, 38*) is used to understand the HIV infection dynamics as:

$$\begin{aligned} \frac{dT}{dt} &= \lambda - dT - kVT \\ \frac{dI}{dt} &= kVT - \delta I \\ \frac{dV}{dt} &= \pi I - cV \end{aligned} \tag{S75}$$

where includes activated CD4+ T cells (T), productively infected CD4+ T cells (I), and HIV concentration (V). The following details regarding the model parameters are summarized in Table S1. To account for the latency between exposure and clinical observation, the interval from the initial infection event to the first viral load measurement is fixed at 35 days (*38*).

Spatiotemporal dynamics of $A\beta$ on the brain

The spatiotemporal dynamics of $A\beta$ on the brain is established by Network-based PDE model as

$$\frac{d\boldsymbol{A}}{dt} + L\boldsymbol{A} = \boldsymbol{\lambda} \odot \boldsymbol{A} \odot (\boldsymbol{K} - \boldsymbol{A}). \tag{S76}$$

Where $\boldsymbol{A} \in \mathbb{R}^{68}, \boldsymbol{\lambda} \in \mathbb{R}^{68}$, and $\boldsymbol{K} \in \mathbb{R}^{68}$ represent the $A\beta$ concentration, recruitment rate of $A\beta$, and capacity of $A\beta$, respectively. L is the patient-specific graph Laplacian corresponding to the patient's specific brain functional connectivity network. We use the functional connectivity (FC) matrix of 68 regions to compute the patient-specific graph Laplacian L, which refers to the our group previous work on how to learn patient-specific functional connectivity matrix (*41*).

Supplementary Figures

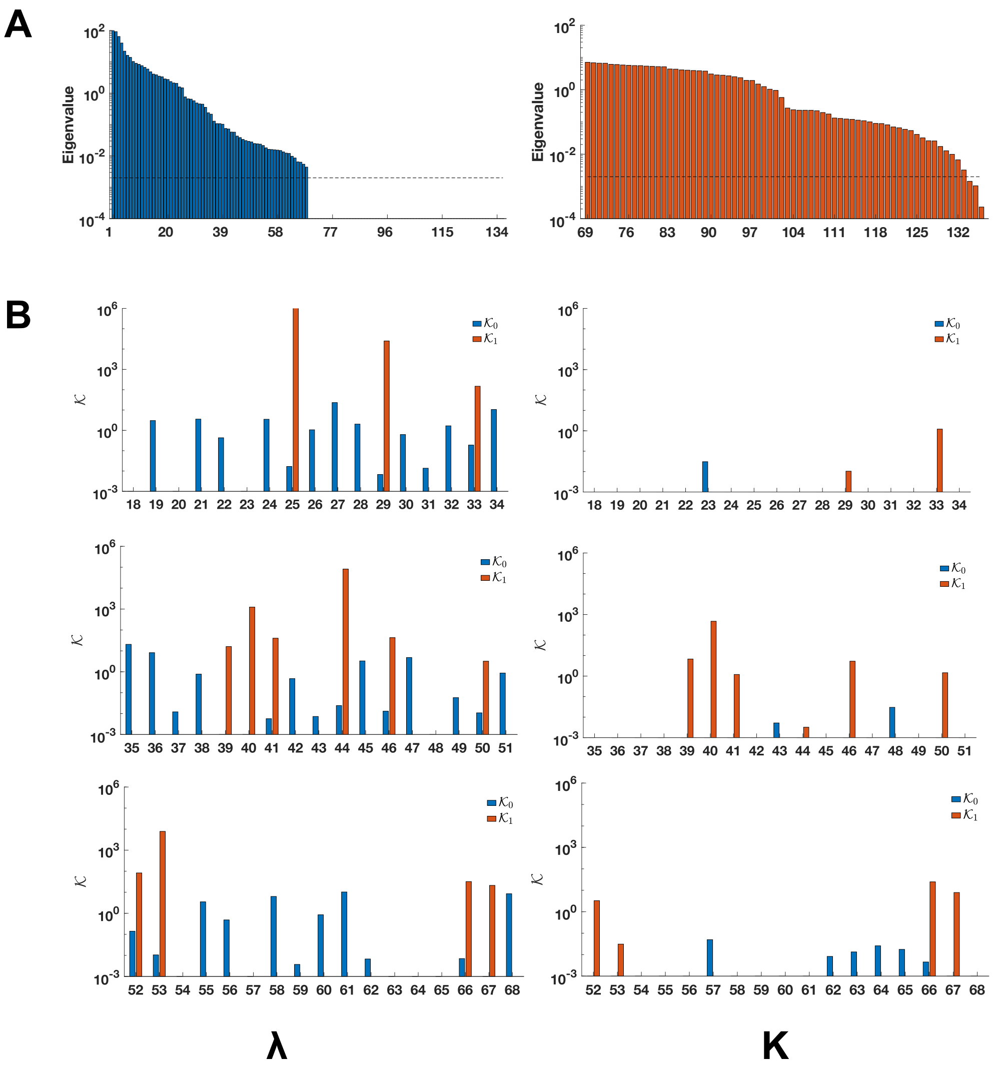

Figure S1: **Validation method accuracy in polynomial fitting.** **(A)** Eigenvalue of ε-order practical identifiability analysis and heatmap of the eigenvector matrix. The dashed line is the threshold $\epsilon = 10^{-3}$. **(B)** The metrics $\mathcal{K}_i$ for conducting practical identifiability analysis for the remaining 51 brain regions.

Supplementary Tables

Table S1: Parameters on the HIV infection Dynamics in Fig. 3.

**Parameter**	**Biological Description**	**Value**	**unit**
λ	the rate of activated CD4+T cells generation	0.0659	$\text{cell} \cdot \text{day}^{-1}$
d	the death rate of activated cells	0.0145	day^{-1}
k	the product rate of activated cell density and virus concentration	0.0008	$\mu\text{l viron} \cdot \text{day}^{-1}$
δ	the death rate of productively infected cells	0.3417	day^{-1}
π	the rate of producing virions	620	$\text{viron} \cdot \text{day}^{-1}$
c	the cleared rate of virus	3.0	day^{-1}

Table S2: Parameters on $A\beta$ Spatiotemporal Dynamics.

**Region Number (i)**	1	2	3	4	5	6	7	8	9	10
$\lambda_i(\times 10^{-2})$	4.83	2.24	2.79	7.63	7.53	0.032	0.004	5.44	0.93	1.72
K_i	5.99	4.50	5.38	0.0005	0.14	0.019	1.11	0.0005	5.96	5.44

Table S3: Parameters on $A\beta$ Spatiotemporal Dynamics.

**Region Number (i)**	11	12	13	14	15	16	17	18	19	20
$\lambda_i(\times 10^{-2})$	11.84	26.02	5.19	112.48	0.45	20.17	3.74	12.29	1.2	59.39
K_i	0.02	0.0001	4.95	0.0061	3.67	0.0044	5.54	0.0141	5.58	0.0069

Table S4: Parameters on $A\beta$ Spatiotemporal Dynamics.

**Region Number (i)**	21	22	23	24	25	26	27	28	29	30
$\lambda_i(\times 10^{-2})$	3.03	5.70	151.19	4.94	0.0005	17.88	2.90	0.22	0.028	2.69
K_i	5.01	3.94	0.0005	5.41	1.32	4.73	4.39	5.71	0.02	4.43

Table S5: Parameters on $A\beta$ Spatiotemporal Dynamics.

**Region Number (i)**	31	32	33	34	35	36	37	38	39	40
$\lambda_i(\times 10^{-2})$	9.20	2.24	3.05	2.25	4.94	2.13	12.44	1.41	17.61	21.12
K_i	0.001	5.85	0.034	5.29	5.34	3.99	0.0055	4.75	0.079	0.004

Table S6: Parameters on $A\beta$ Spatiotemporal Dynamics.

**Region Number (i)**	41	42	43	44	45	46	47	48	49	50
$\lambda_i(\times 10^{-2})$	7.22	0.025	35.61	0.009	4.91	14.91	2.21	152.87	3.82	28.97
K_i	0.0136	3.45	0.013	0.0004	5.357	0.005	4.909	0.0017	0.0085	0.0063

Table S7: Parameters on $A\beta$ Spatiotemporal Dynamics.

**Region Number (i)**	51	52	53	54	55	56	57	58	59	60
$\lambda_i(\times 10^{-2})$	5.77	6.55	0.064	29.03	1.85	11.02	198.06	2.70	19.04	17.28
K_i	5.324	0.0739	0.1337	0.0043	5.296	3.82	0.0019	5.681	0.0138	4.712

Table S8: Parameters on $A\beta$ Spatiotemporal Dynamics.

**Region Number (i)**	61	62	63	64	65	66	67	68
$\lambda_i(\times 10^{-2})$	0.81	45.39	82.04	104.90	69.85	37.93	16.63	0.55
K_i	2.55	0.006	0.0014	0.0034	0.0066	0.006	0.0811	5.87
